# ATPase copper transporter A, negatively regulated by miR‐148a‐3p, contributes to cisplatin resistance in breast cancer cells

**DOI:** 10.1002/ctm2.19

**Published:** 2020-04-07

**Authors:** Ze Yu, Weifan Cao, Yuan Ren, Qijia Zhang, Jia Liu

**Affiliations:** ^1^ Institute of tumor immunology Affiliated Tumor Hospital Guangzhou Medical University Guangzhou China; ^2^ College of Life Science Northeast Forestry University Harbin China; ^3^ Pediatric Laboratory First affiliated Hospital of Harbin Medical University Harbin China; ^4^ Hepatobiliary Internal Medicine Zhuhai Integrated Traditional Chinese and Western Medicine Hospital Zhuhai China; ^5^ School of Pharmaceutical Sciences (Shenzhen) Sun Yat‐sen University Shenzhen China

**Keywords:** ATP7A, breast cancer, chemoresistance, cisplatin, miR‐148a‐3p

## Abstract

**Background:**

Breast cancer is the leading cause of death among women. Cisplatin is an effective drug for breast cancer, but resistance often develops during long term chemotherapy. While the mechanism of chemotherapy resistance is still not fully understood.

**Methods:**

Survival analyses of ATP7A and ATP7B were used to evaluate their effects on the development of Breast invasive carcinoma (BRCA). Immunostaining, flow cytometry, and IC50 assay were utilized to examine the effects of ATP7A‐siRNA combined with cisplatin on apoptosis in breast cancer cells. Q‐PCR, western blotting, and dual‐luciferase assay were employed to confirm ATP7A is a novel target gene of miR‐148a‐3p.

**Results:**

In this current study, we identified knocking‐down ATP7A could enhance cytotoxicity treatment of cisplatin in breast cancer cells. We also demonstrated miR‐148a‐3p overexpression in BRCA cells increased the sensitivity to cisplatin, and subsequently enhanced DNA damage and apoptosis. Moreover, we found ATP7A is a novel target gene of miR‐148a‐3p. In brief, our results showed miR‐148a could accelerate chemotherapy induced‐apoptosis in breast cancer cells by inhibiting ATP7A expression.

**Conclusions:**

Our results highlight that inhibition of ATP7A is a potential strategy for targeting breast cancer resistant to cisplatin, and we provided an interesting method to compare the involvement of various genes in the assessment of cisplatin resistance.

**Abbreviations**
ALCAM: activated leukocyte cell adhesion molecule
ATM: ATM serine/threonine kinase
ATOX1: antioxidant 1 copper chaperone
ATP7A/B: ATPase copper transporting alpha
BCL2: BCL2 apoptosis regulator
BCL2A1: BCL2 related protein A1
BID: BH3 interacting domain death agonist
BRCA1/2: BRCA1/2 DNA repair associated
CASP‐3: caspase 3
CCS: copper chaperone for superoxide dismutase
COX17: cytochrome c oxidase copper chaperone COX17
HSP70: heat shock protein 70
miRNA: microRNA
NRP1: neuropilin 1
SLC31A1: solute carrier family 31 member 1
SMAD2: SMAD family member 2
SPIN1: spindlin 1
TM: Tetrathiomolybdate

## 1 BACKGROUND

Breast cancer is one of the most common malignancies world‐wide for women.^1^, ^2^ It is well demonstrated that genetic alterations are associated with BC development.^3^, ^4^ In China, more than 210,000 new breast cancer cases are diagnosed each year,^5^ with about 5% of which is due to BRCA1/2 mutations.^6^ Basically, molecular subtypes of breast cancer include basal‐like, normal breast‐like, HER2 enriched, luminal A, and luminal B.^7^

Adjuvant chemotherapy is an effective treatment accompanied with surgical in breast cancer patients.^8^ However, chemoresistance is a major impediment for effective BRCA cure.^9^, ^10^ Platinum drugs such as cisplatin and nedaplatin serve as conventional treatment for BRCA and other solid tumors. Among them, cisplatin is widely utilized in advanced breast cancer treatment.^11^ Cisplatin, an effective drug employed to solid cancer treatment, is a DNA‐damaging agent that causes cell death by adduct‐formation.^12^ However, the efficacy of cisplatin is frequently compromised by the development of drug resistance and the insensitivity of malignant cells towards drug treatment, approximately 50 % of cases will develop rapidly acquired drug resistance.^13^ The underlying mechanism of cisplatin resistance is multifactorial, including increased DNA‐repair, alteration of apoptosis signals, and changes in drug transporters.^14^, ^15^, ^16^ The top priority in BRCA is developing effective therapies against chemoresistance, and identification of chemoresistance associated targets is critical for the successful therapies of BRCA. Drug resistance is associated with lots of different physiological changes, mainly including decreased influx and increased efflux of cisplatin.^17^, ^18^ Consequently, future studies on the precise molecular mechanisms of cisplatin sensitivity are required to meet current clinical requirements.

Reduced drug accumulation is the most universally identified correlate of acquired cisplatin resistance.^19^, ^20^ ATPase copper efflux transporter (ATP7A/B) are intracellular copper transporters that maintain cellular copper homeostasis.^21^ Recent evidences suggest ATP7A and ATP7B play fatal roles in drug resistance of platinum. They sequester platinum into vesicular and prevent its cytotoxicity, thus to exercise an indirect effect on platinum‐resistance.^22^, ^23^ Furthermore, Komatsu et al reported that Cu transporters are associated with cisplatin sensitivity. They noted that cells engineered to express high levels of ATP7B were resistant to cisplatin.^24^ Additional evidences implicated ATP7A and ATP7B were found to be overexpressed in some cancer cells with acquired cisplatin resistance.^25^, ^26^

MicroRNAs are a large family of endogenous noncoding RNAs, which are widely found in both plants and animals and involved in various processes including cell proliferation, differentiation, and metabolism.^27^ miR‐148a has common functions of many miRNA species and is implicated in a series of biological processes. miR‐148a is reduced in various cancers and served as a tumor suppressor with crucial roles in cancer progress.^28^ For instance, some studies demonstrated the miR‐148a‐mediated decrease of NRP1 and SMAD2 inhibited cell proliferation and invasion of BRCA cells.^28^, ^29^ Apart from these, miR‐148a was declared to play an essential role in tumor chemoresistance.^30^

This study was designed to explore the role of ATP7A and miR‐148a in mediating resistance to cisplatin in BRCA cells. In this study, we discovered high expression of ATP7A was significantly associated with worse overall survival in BRCA patients. In contrast, ATP7B expression did not display a statistically significant relate to patient prognosis. Then, we confirmed that knocked‐down ATP7A markedly inhibited cell proliferation under cisplatin treatment and promoted cisplatin‐induced apoptosis in BRCA cells. Here, we showed miR‐148a‐3p as a tumor suppressor, and ATP7A as potential target of miR‐148a involved in its cisplatin chemoresistance. Additionally, overexpression of ATP7A markedly reversed miR‐148a‐3p combined with cisplatin induced apoptosis in MDA‐MB‐231 cells. Taken together, these data suggested the miR‐148a‐3p/ATP7A axis which participates in the sensitivity to cisplatin and presents a potential target in breast cancer treatment.

## 2 MATERIALS AND METHODS

### 2.1 Cell lines and cell culture

Breast cancer cell lines T47D and MDA‐MB‐231 were obtained from ATCC. T47D and MDA‐MB‐231 cells were cultured in RPMI‐1640 (Hyclone, Logan, UT). Media were supplemented with 10% fetal bovine serum (FBS; Gibco, NY), 2 mM l‐glutamine (Hyclone), and 1% penicillin/streptomycin (100 U/mL; Hyclone). The cells were cultured at 37°C in a humidified atmosphere of 5% CO_2_. Cisplatin were purchased from sigma (MO).

### 2.2 Cell transfection

For transient transfection of miRNAs, Cells were seeded at 1 × 10^5^ per well in 12‐well plates and cultured for 24 h. Cells were transfected with miR‐148a‐3p mimics (GenePharma, Shanghai, China), or the negative controls at a final dose of 25, 50 nM, using Lipofectamine RNAiMAX transfection reagent (Invitrogen, USA) and in serum‐free Opti‐MEM medium (GIBCO, USA). After 6 h, the medium was replaced with RPMI containing 1% penicillin/streptomycin and 10% FBS.

For silencing specific gene expression, the synthesis of siRNAs was completed by GenePharma company, and transfection of siRNAs targeting ATP7A, ALCAM, NRP1, SMAD2, and SPIN1, as well as nontargeting control was carried out according to the previous protocol of miR‐148a‐3p. Total RNAs and proteins were extracted after 48 h of transfection. Knockdown efficiency of ATP7A was measured using Q‐PCR and western blot. While, the knockdown efficiency of other genes was assessed by PCR.

For enforced expression of ATP7A, ATP7A open reading frame was amplified to the pcDNA3.1(+) expression vector, and the empty plasmid pcDNA3.1(+) was used as a control. Lipofectamine 2000 (Invitrogen) was utilized to transfect the above plasmids into cells. The sequences of siRNA or detection primers are listed in Supporting Information Materials and Figure legends.

### 2.3 Cell viability assay

Cell viability was performed with a CCK‐8 assay (CCK‐8; Beyotime, Shanghai, China). For cell chemosensitivity assay, MDA‐MB‐231 or T47D cells were plated in 96‐well plates (5000 cells per well) for 24 h in triplicate and incubated in a 5% CO_2_ incubator at 37°C. Cell viability was detected as previously described.^31^ Briefly, the cells were transfected with miR‐148a‐3p mimics or si‐ATP7A under a series of doses of cisplatin (0, 15, 30, 45, and 60 μM) following an incubation. Forty‐eight hour post transfection, the cells were incubated at 37°C for 2‐4 h after 10 μL CCK‐8 solution was added to each well. The absorbance at 570 nm (OD value) was measured with a microplate reader (sunrise TECAN, JAPAN).

### 2.4 Cell invasion assay

Twenty‐four‐well plates with polycarbonate membrane inserts (pore size, 8.0 μm; Corning, NY) were used to test cell invasion. The cells (1 × 10^5^) were seeded without serum into 24‐well insert transwell chambers and pretreated with Matrigel (BD, Bioscience, CA). After 48 h of incubation, the migrated cells on the lower surface of the membrane insert were fixed with 4% PFA in PBS, and stained with 0.1% crystal violet and the non‐migratory cells that remained on the upper surface were removed with a cotton swab. The number of cells in the lower chamber was counted with light microscopy.

### 2.5 Measurement of apoptosis by flow cytometry

The apoptotic rate of cells was performed as previously described.^31^ Briefly, analysis of cell apoptosis measured using Annexin V‐PI kit (Beyotime) according to the manufacturer's instructions. Cells were cultured and treated in the same way as cell viability assay. After 48 h, the MDA‐MB‐231 cells were washed twice with ice‐cold PBS and incubated in a buffer supplemented with 5 μL Annexin‐V and 10 μL PI for 40 min in the dark. Finally, these MDA‐MB‐231 cells were analyzed through flow cytometry (BD C6 Biosciences, San Jose, CA).

### 2.6 Measurement of apoptosis by caspase‐3 activated probe

GreenNuc™ Caspase‐3 Assay Kit (Beyotime) is a new‐type of Caspase‐3 green fluorescent substrate with cell membrane permeability to measure the activity of Caspase‐3 in living cells, which can be used to detect the apoptosis of living cells in real time. GreenNuc/Caspase‐3 substrate is a polypeptide DEVD, coupled with DNA green fluorescent dye. The DEVD in the substrate is the recognition sequence of caspase‐3 and has a large negative charge. These negative charges repel the negative charges carried by the DNA, making the DNA green fluorescent dye in the substrate unable to bind to DNA and thus cannot be excited to produce green fluorescence. When the initially non‐fluorescent substrate passes through the cell membrane into the cytoplasm, it is recognized and splintered by caspase‐3 in apoptotic cells, thus releasing activated DNA green fluorescent dye molecules. When this DNA green fluorescent dye migrates into the nucleus and binds to DNA, it can be excited to produce a bright green fluorescence.

The GreenNuc™ Caspase‐3 reagent was directly added to the culture medium and incubated at room temperature for 30 min. Fluorescence microscopy was used for measuring the fluorescence intensity.

### 2.7 RNA extraction and qRT‐PCR analysis

Total RNA was extracted from cultured cells with TRIzol reagent (Invitrogen, Carlsbad, CA) according to the manufacturer's protocol. One microgram of total RNA was reverse‐transcribed with the All‐in‐one First‐Strand cDNA Synthesis SuperMiX kit (TransGen, Beijing, China). The mRNA expression levels of ATP7A, ALCAM, NRP1, SMAD2, and SPIN1 were quantified by real‐time PCR. Primers of these genes were synthesized by GenePharma (Shanghai, China). qRT‐PCR was carried out with SYBR green PCR Master Mix (TransGen) on a Light‐Cycler 480 detection System (Roche, Basel, Switzerland). The relative expression levels of mRNA were calculated by using the 2 −ΔΔCt method. β‐actin was served as internal control.

### 2.8 Immunofluorescence analysis

For immunostaining, cells growing on 12‐well plate were fixed in PFA 4% for 20 min, then washed twice, permeabilized with 0.5% Triton X‐100 for 10 min, washed twice, and blocked in PBS containing 3% bovine serum albumin for 2 h at 4°C. After blocked, cells were incubated with the specific antibodies which were diluted as follows: Caspase‐3 (1:200, ab13847, Abcam, OR) and pH2A.X(S139) (1:200, ab131385, Abcam) overnight at 4°C. Subsequently, cells were incubated with secondary fluorescently antibodies (Alexa fluorophores, Life Technologies) for 1 h at room temperature and DAPI (Beyotime) was applied for 7 min to stain cell nuclei. Images were acquired on a Leica DMR microscope and the colocalization of fluorescent signals were analyzed using ImageJ software.

### 2.9 Western blot analysis

The cells were lysed, and total proteins were extracted and examined for concentration using BCA Protein Assay Kit. Cell lysates were separated by 12% SDS‐PAGE and transferred onto PVDF membranes (Millipore, CA). These PVDF membranes were incubated with the antibodies against Caspase‐3 (Abcam, 1:1000, ab13847), ATP7A (Abcam, 1:1000, ab13995), ATP7B (Abcam, 1:1000, ab124973), and β‐actin (Abcam, 1:1000, ab8227) overnight at 4°C and further incubated with HRP‐conjugated secondary antibody (1:6000).

### 2.10 Luciferase assay

Luciferase assay was performed as previously described.^32^ Briefly, to evaluate the function of miR‐148a, the 3'UTR of ATP7A was amplified and inserted downstream of luciferase reporter gene in the pMIR‐Report luciferase reporter vector (Promega, CA). ATP7A‐WT‐3'UTR vector (Wild‐type ATP7A reporter) and ATP7A‐MUT‐3'UTR vector (mutant ATP7A reporter) were constructed, respectively. The pGL3 renilla luciferase vector was used as internal control. For the luciferase assay, 293T cells were seeded into a 12‐well plate, co‐transfected with the indicated 3'UTR luciferase vectors and miR‐148a‐3p mimic for 48 h. Luciferase activity was then measured by the GloMax‐20/20 Luminometer system from Promega.

### 2.11 Tumorigenicity assay

Female BALB/c nude mice (4‐week‐old) were received from the Beijing Vitalstar Biotechnology Co., Ltd. (Beijing, China) and kept under pathogen‐free conditions in the Laboratory Animal Center. The nude mice were divided into 3 subgroups: the control group (LV‐miR‐nc+PBS); LV‐miR‐nc+cisplatin; LV‐miR‐148a‐3p+cisplatin. Four mice were in each group. A total of 1 × 106 stable MDA‐MB‐231 cells infected with LV‐miR‐NC or LV‐miR‐148a‐3p in 100 μl of PBS were inoculated subcutaneously on the back of the right hind leg. One week after injection, we intraperitoneally injected the mice with suspensions of PBS containing cisplatin (5 mg/kg) or PBS alone three times per week. After 24 days, the mice were killed and the tumors excised, followed by weighing.

### 2.12 Database analyses

Correlation analysis of ATP7A and ATP7B came from ENCORI,^33^ cBioPortal,^34^ linkedOmics,^35^ and GEPIA.^36^ Differential expression analysis of ATP7A and ATP7B with different tumor histological types for BRCA was from LinkedOmics; the survival curves were analyzed using data from the GEPIA, linkedOmics, UCSC Xena,^37^ and KMplot^38^ website. The survival biomarkers analysis (ATP7A/B) for cancer outcomes in risk groups were from SurvExpress.^39^ Enrichment analysis of ATP7A or ATP7B related genes was from linkedOmics. Hsa‐miR‐148a‐3p negatively correlated genes (RNAseq & Proteome) also came from linkedOmics. Prediction of miRNA (miR‐148a) targets was using Targetscan, TarBase v.8, miranda, and miRDB. Methylation analysis of ATP7A was also from UCSC Xena.^40^ GEO database and CCLE provides the expression levels of ATP7A and miR‐148a‐3p in BRCA patients and cells, respectively.^41^ Protein‐drug Correlation Analysis dataset was obtained from TCPA‐portal (MCLP).^42^

All bioinformatics database websites are shown in Supporting Information Materials and Figure legends.

### 2.13 Statistical analysis

Statistical analysis was performed using GraphPad Prism5 software. All data were shown as the mean ± standard deviation (SD) and carried out by three independent experiments. The Student's *t*‐test was used for comparison between two groups. Correlation between genes was evaluated using Spearman's test. Values of ^*^ *P* < .05, ^**^ *P* < .01, and ^***^ *P* < .001 were considered statistically significant.

## 3 RESULTS

### 3.1 Compared with ATP7B, ATP7A is a potential oncogene in breast cancer

**FIGURE 1:**
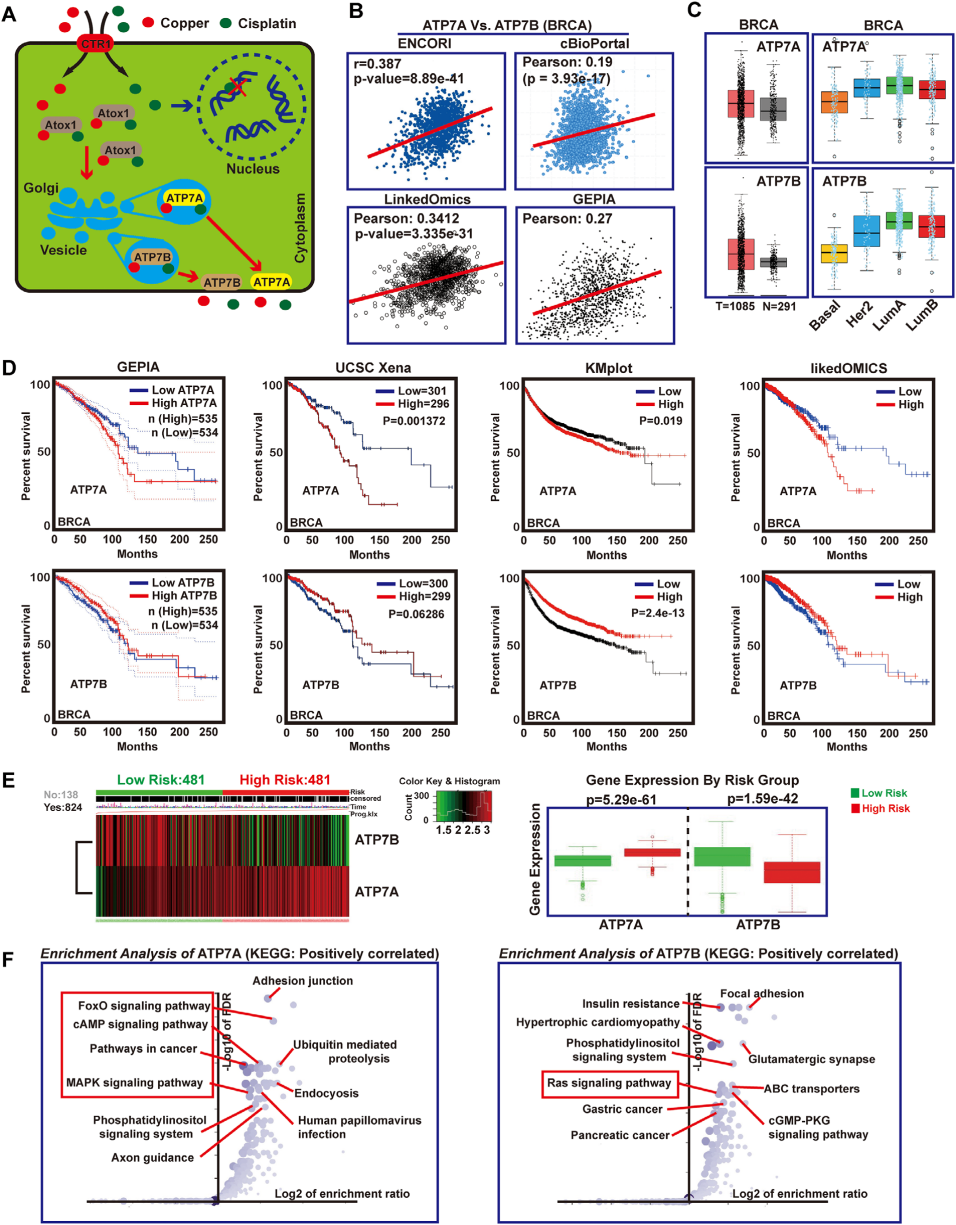
ATP7A is a potential oncogene in breast cancer. A, The schematic model of ATP7A/ATP7B regulation of cancer cell cisplatin‐resistance. B, Pearson correlation analysis between mRNA levels of ATP7A and ATP7B in BRCA samples (data came from ENCORI, cBioPortal, LinkedOmics and GEPIA). C, Analysis of ATP7A and ATP7B expression in BRCA and corresponding normal tissues using GEPIA and linkedOmics database. D, Survival curves analysis for breast cancer patients with low versus high expression levels of ATP7A and ATP7B. E, Heat map shows the expression of ATP7A and ATP7B along samples in risk groups. Low expression is represented in green grades and high expression in red grades; box plots shows the expression of ATP7A and ATP7B genes in risk groups, including the *P*‐value testing for difference using *t*‐test. F, Enrichment analysis of ATP7A and ATP7B related genes, respectively

Although ATP7A and ATP7B were reported to be involved in cisplatin‐resistance, the mechanisms were still unknown. It has been demonstrated that cross‐resistance between platinum and copper was bidirectional.^43^ Thus, copper and cisplatin may share the same Cu transporters system (Figure 1A). Platinum drugs along the Cu‐transport pathways may function as a mechanism of drug delivery, which could explain the relevance between ATP7A/B and cisplatin resistance.^44^ As we know, ATP7A is correlated with cisplatin resistance in different cancers, and similar to ATP7A, ATP7B mediates resistance to cisplatin in various tumors.^45^ However, which ATPase is associated with cisplatin resistance is unknown in breast cancer.

In this study, to compare the difference between ATP7A and ATP7B in cisplatin resistance, we first analyzed the correlation between ATP7A and ATP7B in BRCA. Data from four databases showed a significant positive correlation between ATP7A and ATP7B in BRCA (Figure 1B). Furthermore, the expression levels of ATP7A and ATP7B in BRCA tissues were slightly higher than those in normal tissues, and the expression of ATP7A and ATP7B in different subtypes of breast cancer was also similar (Figure 1C). However, it is interesting that statistics from four survival analysis databases showed high expression level of ATP7A was significantly associated with worse overall survival in BRCA patients, whereas ATP7B did not display a statistically significant association with patient prognosis (Figure 1D). These results illustrated potential importance of ATP7A for breast cancer. Meanwhile, SurvExpress analysis results indicated that ATP7A were able to separate risk groups characterized by differences in its gene expression, the expression level of ATP7A was significantly increased in the high risk of recurrence (Figure 1E). Enrichment analysis of genes positively correlated with ATP7A showed that these genes were involved in a variety of proliferative signaling pathways, while ATP7B‐related genes did not show this trend (data came from linkedOmics) (Figure 1F), more difference analysis between ATP7A and ATP7B can be seen in Supporting Information File 1A‐J. These above results suggested ATP7A, rather than ATP7B, played an important role in the development of breast cancer.

### 3.2 ATP7A enhances breast cancer cells resistance to cisplatin

**FIGURE 2:**
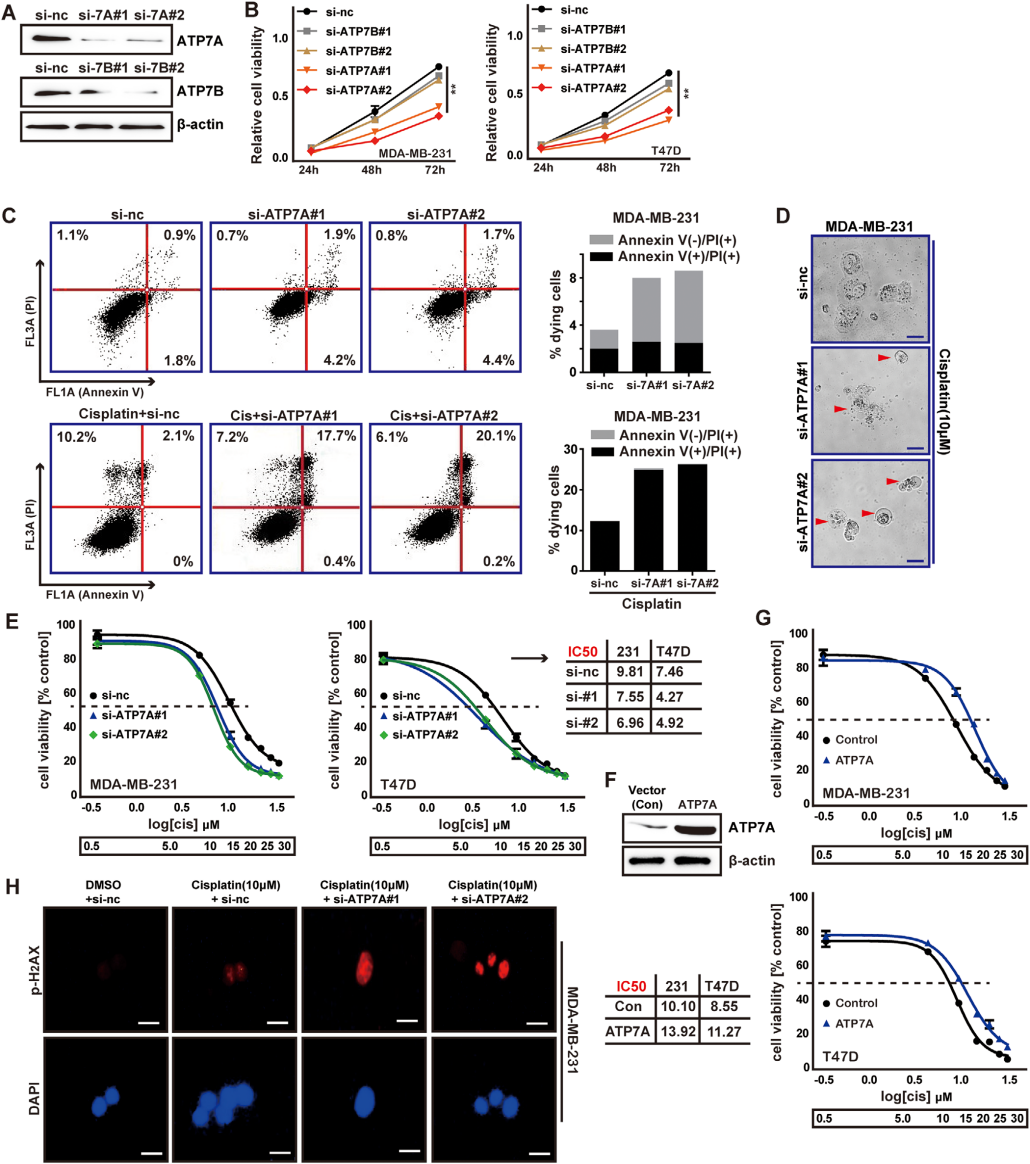
ATP7A induces cisplatin resistance in breast cancer cells. A, MDA‐MB‐231 cells were transfected with ATP7A or ATP7B siRNAs, and ATP7A or ATP7B expression was examined by western blot. B, Cell viability was determined using CCK‐8 in T47D and MDA‐MB‐231 cells transfected with si‐ATP7As or si‐ATP7Bs. C, Flow cytometry analysis of apoptosis rates in MDA‐MB‐231 (with si‐ATP7As) cells with or without cisplatin treatment. D, Cisplatin induces morphology changes in MDA‐MB‐231 cells transfected with ATP7A‐siRNAs. E, Cisplatin IC50s calculated using CCK‐8 in breast cancer cells transfected with si‐ATP7As or si‐nc. F, Western blot was used to detect overexpression of ATP7A. G, Cisplatin IC50s calculated using CCK‐8 in cells treated with overexpressed ATP7A. H, Immunofluorescence of *P*‐H2AX was used to evaluate the degree of DNA‐damage in cisplatin‐treated MDA‐MB‐231 cells transfected with ATP7A‐siRNAs. Each bar in the figure represents the mean ± SEM of triplicates. ^**^ *P* < .01

To explore the cisplatin‐sensitivity of breast cancer cells knocked‐down ATP7A by siRNA, first, MDA‐MB‐231 cells were transfected with si‐ATP7A to achieve ATP7A knockdown, as verified using western blot (Figure 2A). Then, we investigated the roles of ATP7A and ATP7B in proliferation of breast cancer cells by transfecting ATP7A or ATP7B siRNAs into MDA‐MB‐231 and T47D cells, respectively. Results of CCK‐8 assays showed the cell viability was inhibited by si‐ATP7As, compared to the si‐nc group, whereas ATP7B‐siRNAs did not cause significant changes (Figure 2B). We further analyzed the effects of ATP7A know‐down on apoptosis of MDA‐MB‐231 cells under cisplatin treatment by Annexin V/PI staining. We observed, compared with si‐nc group, MDA‐MB‐231 transfected with ATP7A‐siRNAs showed increased apoptosis under cisplatin treatment. In addition, down‐regulation of ATP7A alone did not induce significant apoptosis (Figure 2C,D). Meanwhile, the same treatment for ATP7B could not achieve the same phenotypes of ATP7A (Supporting Information File 2A). As shown in Figure 2E, ATP7A knockdown conferred weaker cisplatin‐resistance to MDA‐MB‐231 cells in the IC50 values. Overexpression of ATP7A in MDA‐MB‐231 cell significantly increased the IC50 value (Figure 2F,G). These findings were further confirmed in T47D cells (Figure 2E‐G).

Next, we assessed whether breast cancer cells knocked‐down ATP7A with cisplatin treatment produced more DNA damage than cisplatin alone. IF analysis of p‐H2AX showed increased DNA damage after ATP7A‐siRNA combined with cisplatin, compared to cisplatin alone (Figure 2H). Taken together, these results indicated that the combination with knocked‐down ATP7A and cisplatins induced apoptosis in MDA‐MB‐231 and T47D cells.

### 3.3 ATP7A‐mediated cisplatin resistance is associated with vesicle transport

**FIGURE 3:**
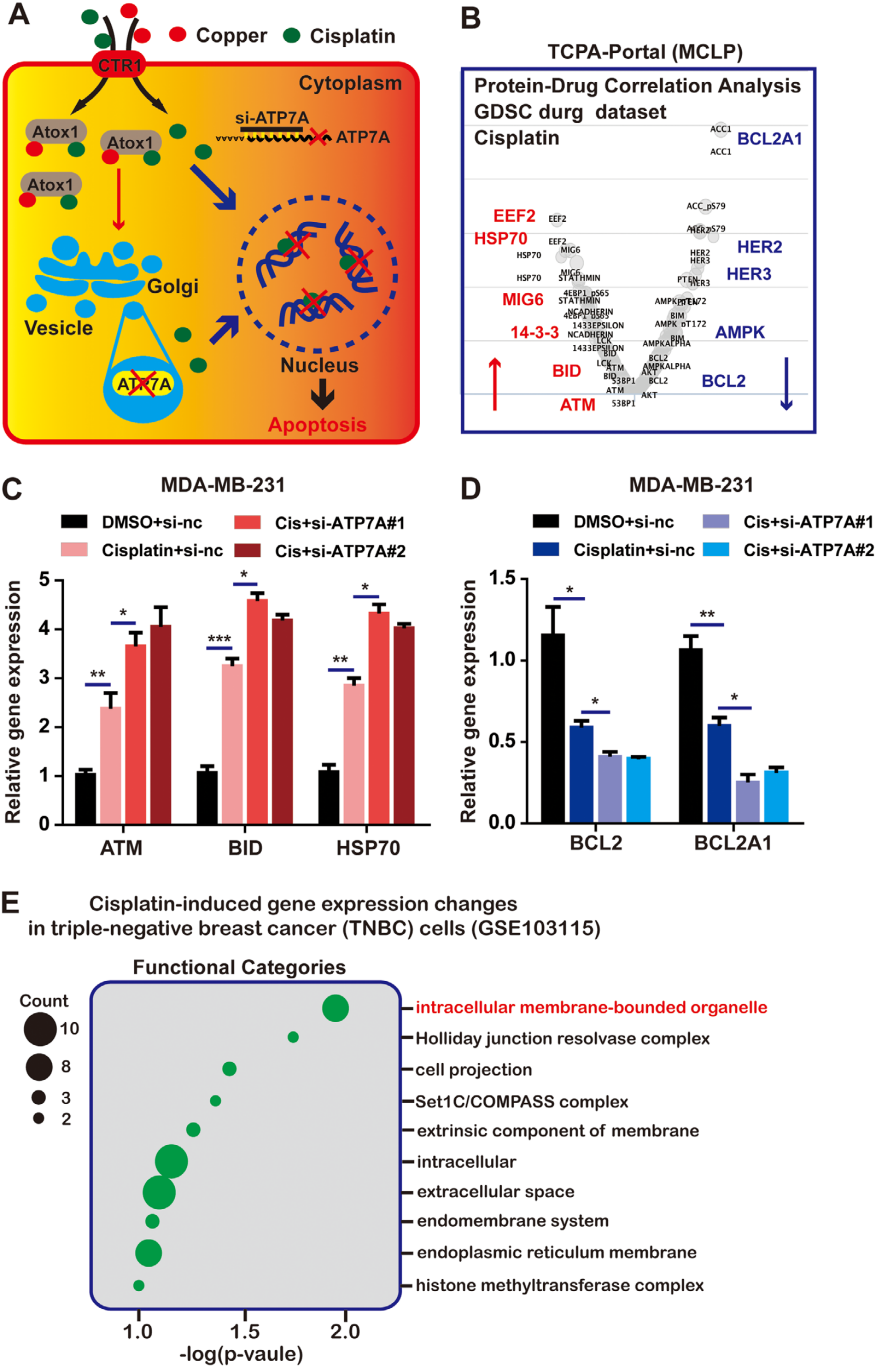
Microarray analysis of TNBC cells treated with cisplatin. A, The schematic model of knocked‐down ATP7A increased cancer cell sensitivity to cisplatin. B, MCLP database showed Protein‐drug (cisplatin) correlation analysis in BRCA. C and D, RT‐PCR analysis of ATM, BID, HSP70, Bcl2 and BclA1 expression levels in cisplatin‐treated MDA‐MB‐231 cells transfected with ATP7A‐siRNAs, compared with the cells treated with cisplatin alone. E, Bubble chart showing the top enriched pathways by DAVID enrichment analysis (functional categories) to demonstrate the difference in gene enrichment in cisplatin‐induced TNBC cells. Each bar in the figure represents the mean ± SEM of triplicates. ^*^ *P* < .05, ^**^ *P* < .01, and ^***^ *P* < .001

As we studied above, knocking‐down ATP7A increased the sensitivity of breast cancer cells to cisplatin (Figure 3A). To further explore the relationship between ATP7A and cisplatin resistance in breast cancer, first, we used the protein‐drug correlation analysis‐function of the MCLP dataset to identify the changing proteins in breast cancer cells due to cisplatin toxicity, results showed that the elevated genes were mainly involved in DNA repair and protein folding, and the decreased genes mainly participated in blocking apoptosis in cisplatin treatment (Figure 3B). Silenced ATP7A in MDA‐MB‐231 cells treated with cisplatin increased ATM, BID, and HSP70 expression, and reduced Bcl2 and Bcl2A1 levels, compared to cisplatin alone (Figure 3C,D). These findings indicated that reducing ATP7A aggravated the cytotoxicity of cisplatin.

To understand the mechanism of cisplatin‐resistance in breast cancer cells, we used the GEO dataSets (GSE103115) to evaluate changes in gene expression profiles in four types of TNBC cells (HCC38, MDA‐MB‐231, BT549 and MDA‐MB‐157) under treatment with cisplatin for 24 hours and 72 hours. As shown in Figure 3E and Supporting Information File 2B, enrichment of functional categories and GO analysis of these differentially expressed genes identified pathways that were significantly changed in cisplatin treated cancer cells. Considering the number of genes and p‐value, these significantly altered genes, including ATP7A, were related to cytoplasmic vesicle transport, and of note, these genes were all upregulated. Subsequent experimental validation also demonstrated that ATP7A showed concentration‐dependent upregulation in cisplatin treated cells (Supporting Information File 2C). Therefore, we speculated that these elevated genes related to vesicle transport may be accountable for the cisplatin resistance phenotypes observed in breast cancer cells.

### 3.4 Cisplatin does not cause changes of intracellular copper levels in breast cancer cells

**FIGURE 4:**
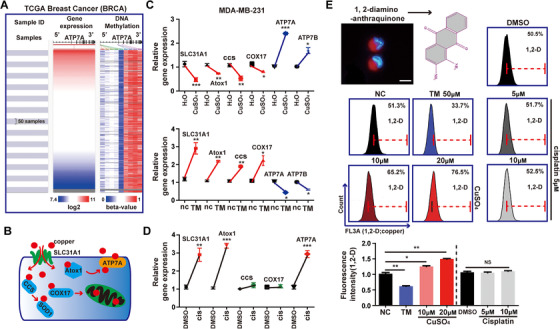
Cisplatin does not affect intracellular copper levels. A, The methylation level of ATP7A in BRCA samples is shown (Column 1 represents BRCA samples, Column 2 represents ATP7A expression, Column 3 represents the DNA methylation clusters in corresponding sample). B, The schematic model of main pathways of intracellular copper metabolism. C, The mRNA expression levels of SLC31A1, Atox1, CCS, Cox17 were decreased in MDA‐MB‐231 cells treated with CuSO4, while ATP7A and ATP7B increased with the same condition. Accordingly, TM led to upregulation of SLC31A1, Atox1, CCS, Cox17 and decrease in ATP7A and ATP7B. D, The mRNA expression levels of SLC31A1, Atox1 and ATP7A were increased in MDA‐MB‐231 cells treated with cisplatin, while CCS and Cox17 showed no significant difference. E, Flow cytometry analysis was performed to detect copper level in cisplatin‐treated MDA‐MB‐231 cells, CuSO4 treatment as a positive control, while TM treatment as a negative control. Each bar in the figure represents the mean ± SEM of triplicates. ^*^ *P* < .05, ^**^ *P* < .01, and ^***^ *P* < .001

Our previous researches have demonstrated that ATP7A is involved in cisplatin resistance in breast cancer. Next, we will explore what factors regulate ATP7A levels and the mechanism of ATP7A upregulation induced by cisplatin in breast cancer. First, as shown in Figure 4A, the UCSC Xena Browser tool was used to analyze the relationship between methylation of ATP7A and ATP7A gene expression in breast cancer. Results from the heap‐map showed that ATP7A expression and its DNA methylation were uncorrelated in same data cohort. In light of the reported, as a major copper transporter, ATP7A, like other copper transporters, is regulated by intracellular copper levels.^46^ In this study, we also uncovered the genes involved in copper absorption and transport (SLC31A1, Atox1, CCS and Cox17) were significantly increased, while copper efflux transporters (ATP7A and ATP7B) (Figure 4B) were markedly decreased when copper levels were elevated in MDA‐MB‐231 cells. Addition of copper chelator TM achieved the opposite effect (Figure 4C).

As mentioned earlier, accumulation and transport of cisplatin in cells are closely related to the delivery of copper.^47^ Subsequently, we verified that copper could reduce the cell sensitivity to cisplatin, while TM in turn could increase the anticancer effect of cisplatin in MDA‐MB‐231 and T47D cells (Additional file 3A‐D).

Based on the above conclusions, we boldly proposed a hypothesis that since the level of copper can modulate the accumulation of cisplatin, in turn, whether cisplatin can affect intracellular copper level, thereby regulating the expression of ATP7A. The data showed that only the genes involved in copper uptake and efflux processes varied significantly, while CCS and COX17 showed no significant difference under cisplatin treatment (Figure 4D). Besides, 1, 2‐diamino‐anthraquinone, act as a fluorescent probe, was used to detect intracellular Cu ions,^48^ and flow cytometry results displayed there was no significant difference in copper level after cisplatin treatment (Figure 4E). Therefore, these results suggested that cisplatin increased “copper flow” but did not result in intracellular copper accumulation.

### 3.5 miR‐148a‐3p targets ATP7A in breast cancer cells

**FIGURE 5:**
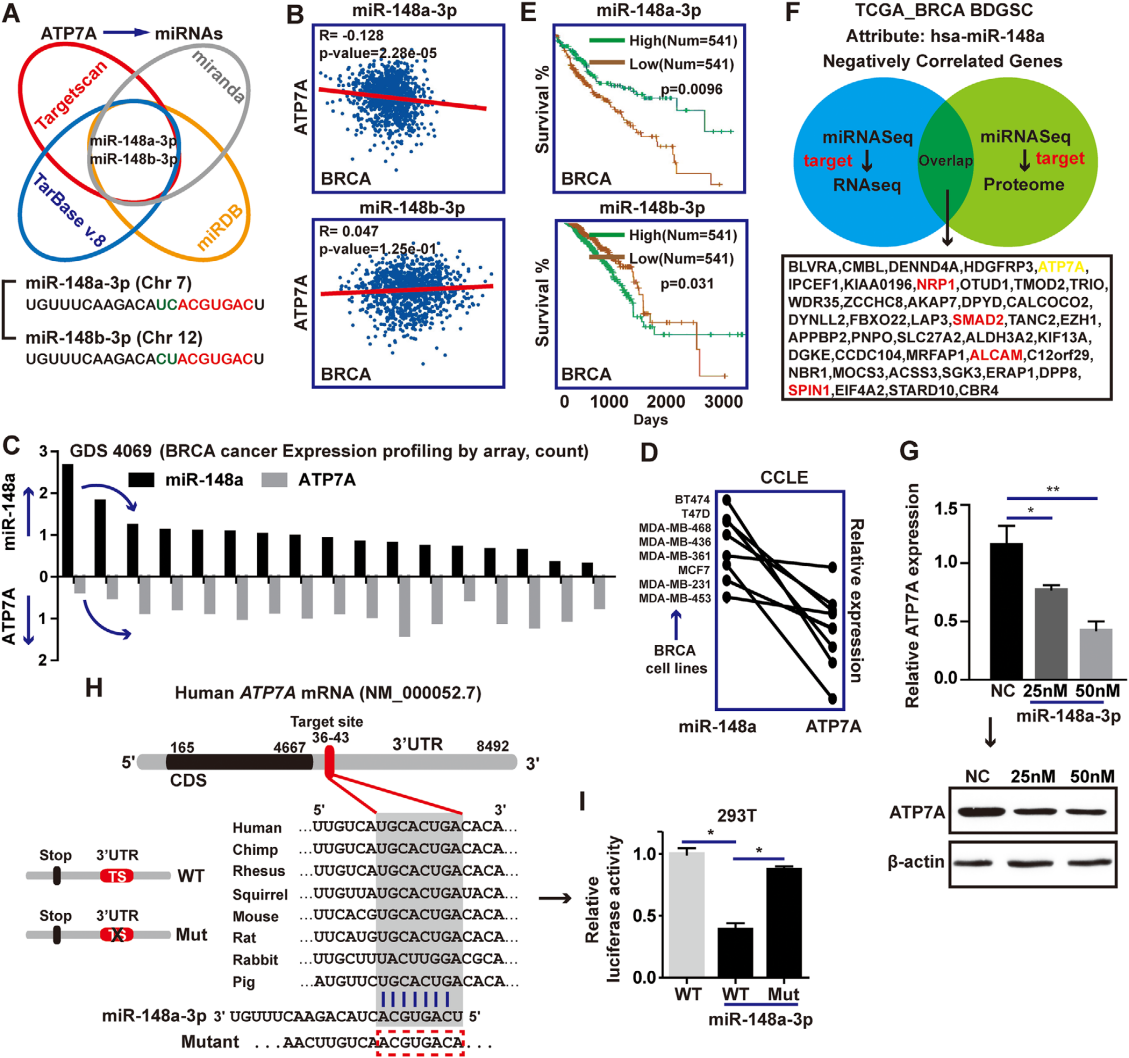
ATP7A is the target of miR‐148a‐3p. A, Venn diagram of putative miRNAs targeting ATP7A. B, Pearson correlation analysis between ATP7A and miR‐148a‐3p in BRCA samples. C and D, Expression analysis of ATP7A and miR‐148a‐3p in the same BRCA samples and cell lines. E, Survival curves analysis for breast cancer patients with high versus low expression levels of miR‐148a‐3p or miR‐148b‐4p. F, Overlap of negative correlation genes and proteins of miR‐148a‐3p. G, After treatment with doses of miR‐148a‐3p mimic for 72 hours, western blot analysis was performed to detect ATP7A protein levels in MDA‐MB‐231 cells. H, Wild‐type ATP7A luciferase reporter vector and mutant‐ATP7A luciferase reporter vector containing an 8 bp mutation in the predicted miR‐148a‐3p binding sites were constructed. I, The relative luciferase activity was significantly reduced in the 293T cells transfected with miR‐148a‐3p and these effects could be abolished by mutation of ATP7A 3'‐UTR. Each bar in the figure represents the mean ± SEM of triplicates. ^*^ *P* < .05, ^**^ *P* < .01

Now, we considered post‐transcriptional regulation of ATP7A, focusing on miRNAs that regulate ATP7A. To search for putative miRNAs that target ATP7A, we used the TargetScan, miranda, miRDB, and TarBase v.8 prediction tools and identified two candidate miRNAs that directly targets the 3'UTR of ATP7A (Figure 5A). Among them, the difference between miR‐148a and miR‐148b is only two bases before the seed sequence. YM500v2 database showed that miR‐148a‐3p was dominant in most human tissues compared with miR‐148a‐5p (Supporting Information File 4A,B).

To further determine the miRNA targeting ATP7A, first, we found miR‐148a‐3p expression was negatively correlated with ATP7A expression, while miR‐148b‐3p expression was positively correlated with ATP7A expression (Figure 5B). Moreover, data from GEO (GDS4069) and CCLE revealed a significantly negative correlation between miR‐148a and ATP7A (Figure 5C,D). Next, we analyzed the prognostic significance of miR‐148a and miR‐148b expression in BRCA patients. Survival curves showed BRCA patients with high miR‐148a expression were associated with high overall survival. However, the functions of miR‐148b in cancers were rarely reported, and there was no significant correlation between its expression level and survival rates of breast cancer patients (Figure 5E). Besides, we profiled the global miRNA levels of metastatic BRCA compared to non‐metastatic BRCA (GSE100453), and found miR‐148a‐3p were significantly downregulated in metastatic BRCA (Supporting Information File 4C). The expression level of miR‐148a was significantly lower than that of miR‐148b in breast cancer cell lines MDA‐MB‐231 and T47D (Supporting Information File 4D), as the same as breast cancer samples (Supporting Information File 4E). These observations also suggested miR‐148a‐3p was a potential tumor suppressor in BRCA. Using the multi‐omics analysis method of linkedOmics database, we found many potential miR‐148b‐3p targets, some of which had been verified in some literatures.^28^, ^29^, ^48^, ^49^ ATP7A as the target in this study also appeared in the prediction data (Figure 5F).

Finally, qRT‐PCR and western blot analysis displayed the ATP7A expression was decreased in MDA‐MB‐231 cells transfected with miR‐148a‐3p in a dose dependent manner (Figure 5G). Besides, transfection with miR‐148a‐3p decreased the luciferase activities of pMIR‐ATP7A (ATP7A‐WT‐3'UTR) plasmids, but not the mutant ATP7A (ATP7A‐MUT‐3'UTR) plasmids (Figure 5H,I). These results, along with the above findings, indicated ATP7A was a functional target of miR‐148a in breast cancer cells.

### 3.6 miR‐148a‐3p promotes cisplatin‐induced apoptosis in breast cancer cells

**FIGURE 6:**
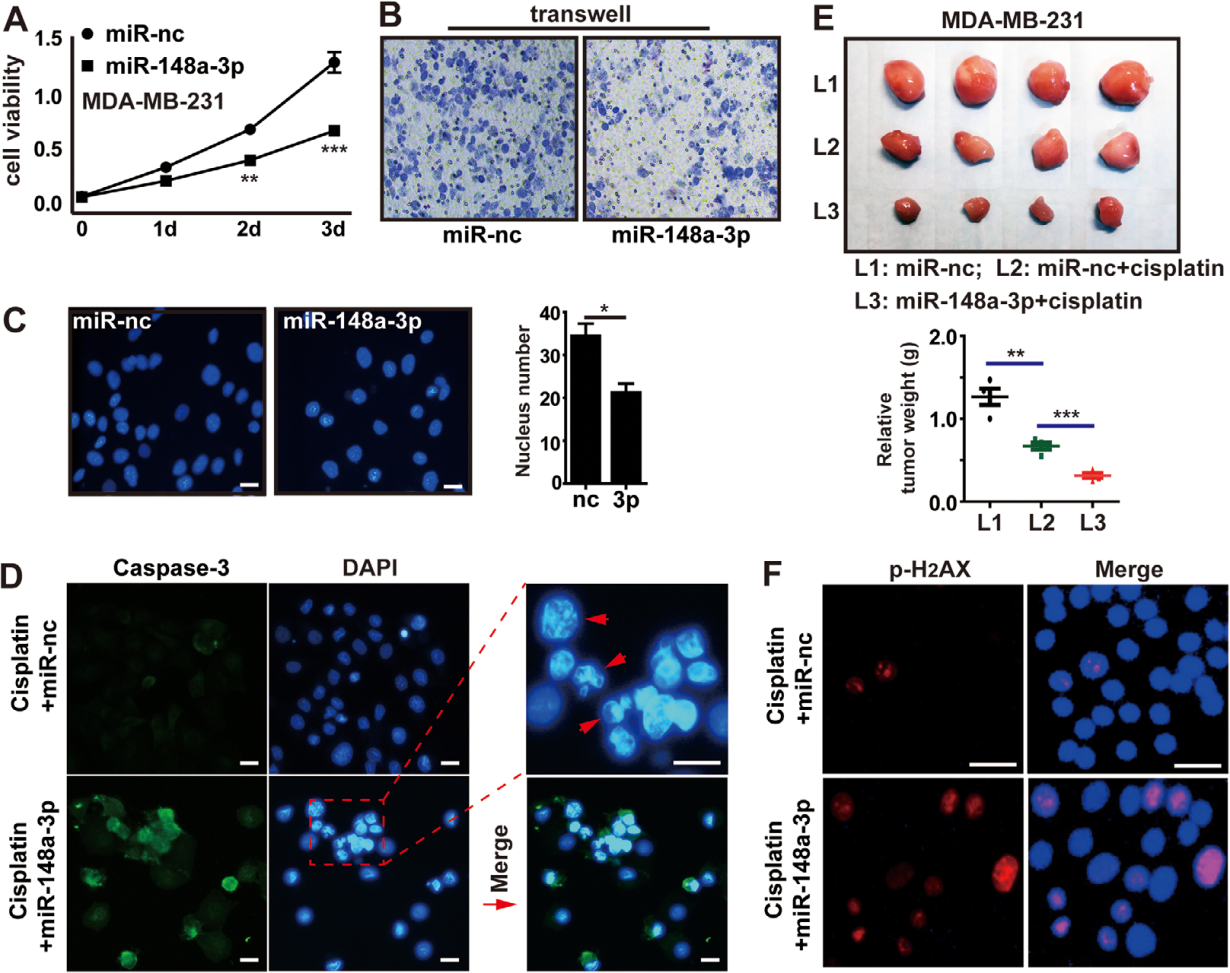
The role of miR‐148a‐3p in breast cancer cell cisplatin‐resistance. A and B, cell viability and invasion assay of MDA‐MB‐231 cells treated with miR‐148a‐3p mimics. C, DAPI nuclear staining and nucleus counting were applied to detect apoptosis and cell proliferation in MDA‐MB‐231 cells transfected with miR‐148a‐3p or miR‐nc. D, Immunofluorescence staining of caspase‐3 in cisplatin‐treated MDA‐MB‐231 cells transfected with miR‐148a‐3p. E, Xenograft tumors of sacrificed mice at end of experiment with or without cisplatin treatment. F, Immunofluorescence staining of p‐H_2_AX in cisplatin‐treated MDA‐MB‐231 cells transfected with miR‐148a‐3p. Each bar in the figure represents the mean ± SEM of triplicates. ^*^ *P* < .05, ^**^ *P* < .01, and ^***^ *P* < .001

As we mentioned, miR‐148a‐3p is a tumor suppressor. In breast cancer, we further confirmed that miR‐148a‐3p could inhibit cell proliferation and migration (Figure 6A,B, Supporting Information File 5A), but not cause cell death (Figure 6C, Supporting Information File 5B,C). In order to assess whether the property of cisplatin‐resistance was in part mediated by miR‐148a‐3p in MDA‐MB‐231 cells, we evaluated the effect of miR‐148a‐3p on cells exposed to cisplatin. Transfection of miR‐148a‐3p may increase the sensitivity of MDA‐MB‐231 cells to cisplatin. Immunofluorescence assays showed that miR‐148a‐3p strongly promoted the cisplatin‐induced apoptosis in MDA‐MB‐231 cells, as assessed by caspase‐3 activity and observation of nuclear morphology (chromatin condensation) (Figure 6D). Consistent with its effects in vitro, miR‐148a‐3p combined with cisplatin remarkably suppressed the growth of xenografted tumors in mice, showing a lower weight than the tumors treated with miR‐nc plus cisplatin or miR‐nc alone (Figure 6E). Moreover, increased p‐H2AX levels also indicated that miR‐148a‐3p deepened the DNA damage caused by cisplatin (Figure 6F).

### 3.7 Cisplatin inhibits miR‐148a‐3p in breast cancer cells

**FIGURE 7:**
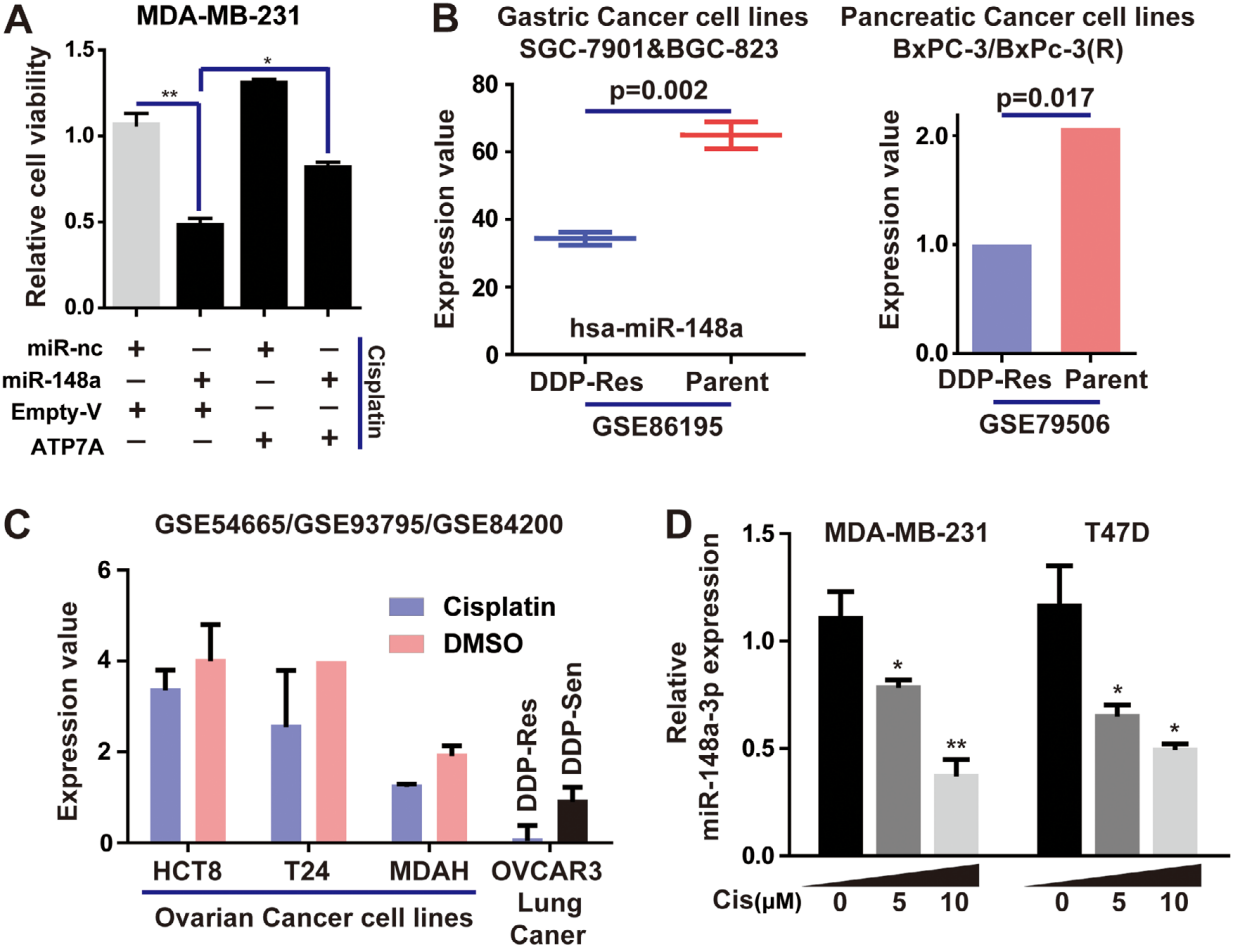
MiR‐148a‐3p restrains cisplatin‐resistance through the regulation of ATP7A in breast cancer cells. A, MDA‐MB‐231 cells were co‐transfected with miR‐148a‐3p or miR‐nc and empty or ATP7A‐overexpressed vectors; cell viability was determined using CCK‐8 assays. B, The expression level of miR‐148a‐3p in cisplatin‐resistant cell lines, compared with normal parent cells. C, The expression of miR‐148a‐3p in cisplatin‐treated cells, compared with cells treated with DMSO. D, Expression of miR‐148a‐3p was determined in MDA‐MB‐231 and T47D cells with the increasing doses of cisplatin for 48 hours. Each bar in the figure represents the mean ± SEM of triplicates. ^*^ *P* < .05, ^**^ *P* < .01

Subsequently, we will explore whether miR‐148a‐3p affects cisplatin resistance of BRCA cells by modulating ATP7A. We demonstrated the effect of miR‐148a‐3p combined with cisplatin on cell viability and apoptosis could be partially reversed by overexpression of ATP7A (Figure 7A, Supporting Information File 5D).

Furthermore, in the above experiments, we proved that ATP7A was the target of miR‐148a‐3p, and overexpressed miR‐148a‐3p increased the sensitivity of breast cancer cells to cisplatin via suppressing ATP7A. Therefore, it was natural to wonder whether cisplatin induced ATP7A expression by reducing the level of miR‐148a‐3p. Based on GEO database (GSE86195, GSE79506, GSE54665, GSE93795, and GSE84200), we found miR‐148a‐3p was significantly downregulated in cisplatin‐resistant cancer cells (gastric cancer and pancreatic cancer), and the level of miR‐148a‐3p also decreased in cisplatin‐treated ovarian and lung cancer cells (Figure 7B,C). In cisplatin‐treated MDA‐MB‐231 and T47D cells, miR‐148a‐3p were significantly downregulated in a dose‐dependent way by qRT‐PCR analysis (Figure 7D).

### 3.8 ATP7A is a major gene involved in cisplatin resistance among the targets of miR‐148a‐3p

**FIGURE 8:**
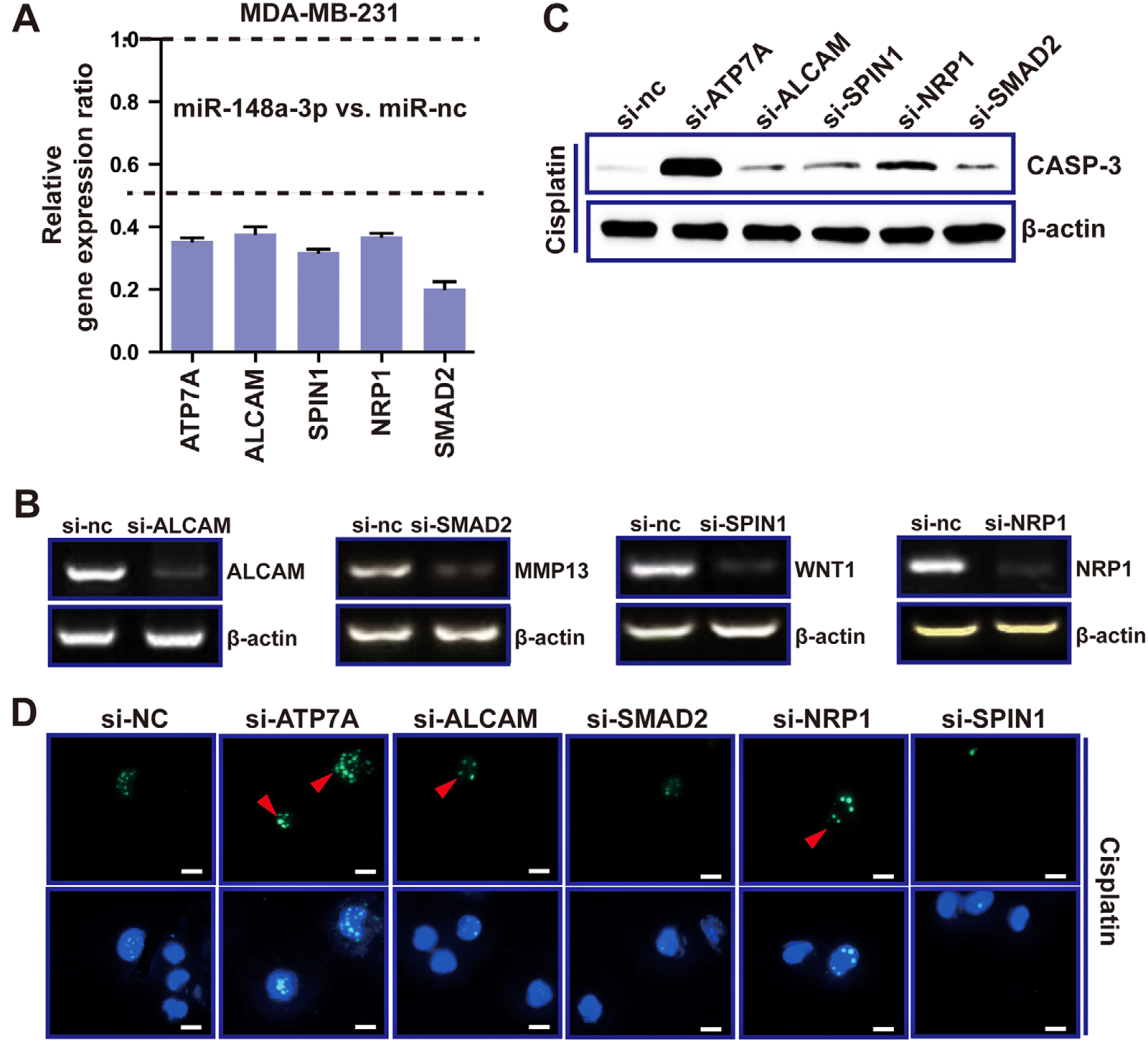
ATP7A is an important target of miR‐148a. A, Expression levels of miR‐148a‐3p targets in MDA‐MB‐231 cells transfected with miR‐148a‐3p mimic were determined by RT‐PCR. B, PCR was used to validate mRNA levels altered through overexpression of the corresponding siRNAs. C, Caspase‐3 activity analysis of MDA‐MB‐231 cells treated with cisplatin and multiple siRNAs, respectively, by western blot. D, Caspase‐3 activated probe was used to detect apoptosis in MDA‐MB‐231 cells treated with cisplatin and multiple siRNAs, respectively

Previously, we found miR‐148a inhibited cell proliferation and inversely regulated ATP7A via direct binding to the ATP7A‐3’UTR. However, as a tumor suppressor in BRCA, miR‐148a‐3p can target multiple genes, which are implicated in oncogenesis, cell adhesion, cell invasion, signal transduction, and metastasis.^28^, ^29^, ^48^, ^49^ We verified these genes were all downregulated in the MDA‐MB‐231 cells transfected with miR‐148‐3p mimic, compared with NC group (Figure 8A). In order to verify whether other miR‐148a‐3p targets besides ATP7A are also involved in cisplatin resistance, first, we searched and designed siRNAs for these genes, and then examined the knockdown effect for these genes (Figure 8B). Next, caspase‐3 activated assay and western blot revealed knocking‐down ATP7A promoted caspase‐3 activation and accumulation induced by cisplatin, but knockdown of other miR‐148a targets did not work as well (Figure 8C,D). These findings suggested ATP7A, as the major target of miR‐148a‐3p, was involved in cisplatin resistance.

## 4 DISCUSSION

**FIGURE 9:**
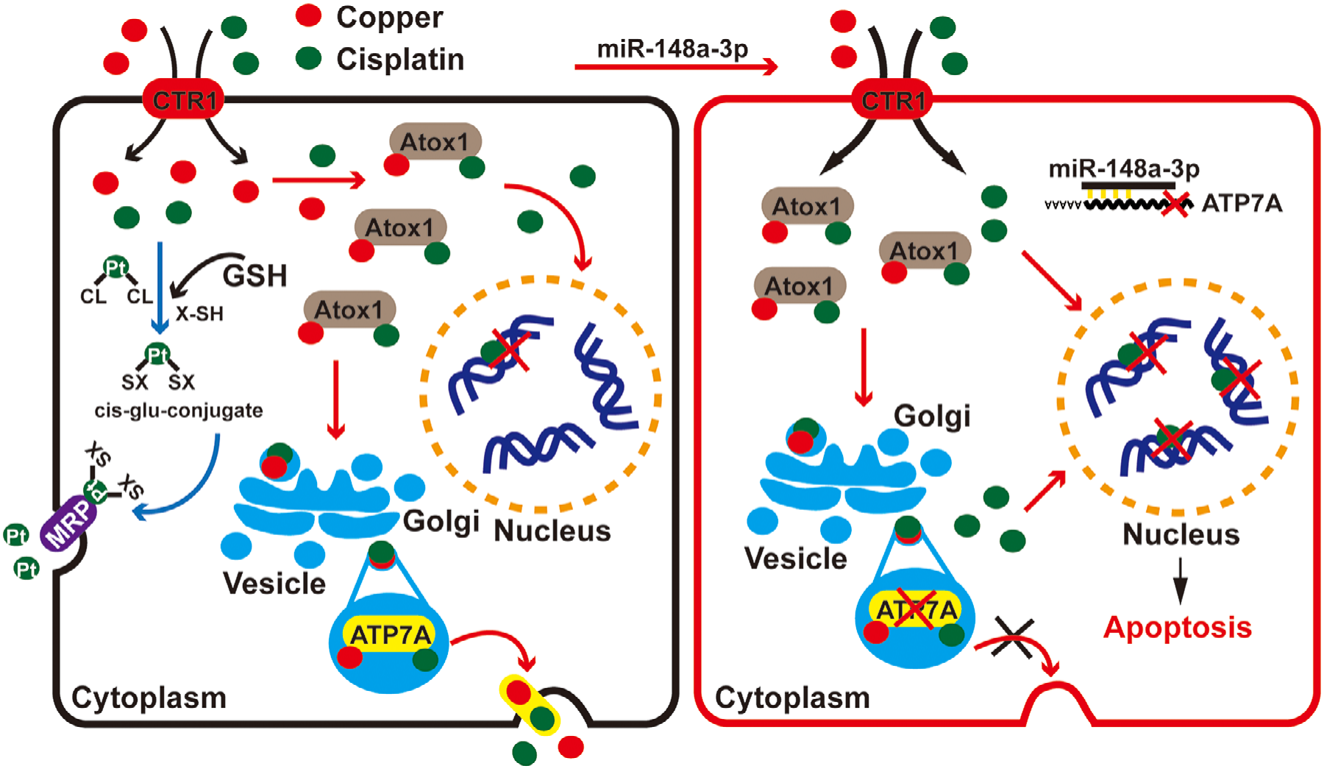
Schematic diagram of the mechanism that ATP7A is involved in cisplatin resistance. The blue arrow represents the efflux of cisplatin through MRPs; the red arrow represents the efflux of cisplatin through ATP7A in our present study

To develop a novel and efficient therapy for BRCA treatment, it is necessary to elucidate the molecular mechanisms underlying drug resistance. The development of drug resistance presents a major impediment to the treatment of breast cancer. Numerous reasons can cause platinum resistance, mainly covering overcome of cytotoxicity mechanisms and the influx and efflux of platinum.^18^, ^50^ Among them, mechanisms of drug efflux are the focus of our attention. It was reported earlier, most studies related to drug resistance are focused on the multidrug resistance associated protein family, which is belonging to the ATP‐binding cassette (ABC) transporters.^51^, ^52^ MRP1 and MRP2 show the ability to strengthen elimination of anticancer drugs. They may enhance drug‐resistance via transporting the cisplatin‐glutathione‐conjugate compounds out of breast cancer cells.^53^ In our study, we found ATP7A and miR‐148a‐3p were involved in cisplatin resistance (Figure 9).

It was reported that dysfunction of ATP7A or ATP7B can make a contribution to dysregulated export of platinum.^23^, ^54^, ^55^ In our study, we focused on elucidation of the biological roles of ATP7A in cisplatin‐resistant treatment. We observed a significant increase in drug efficacy in cisplatin‐resistant breast cancer cells through combining ATP7A‐siRNA with cisplatin. Meanwhile, the breast cancer cells treated with TM, as an active copper chelating agent, could achieve comparable cytotoxicity yielded from ATP7A knockdown. Accumulating lines of evidence suggest that cisplatin enters cells, is distributed to subcellular compartments, and efflux from cells via Cu chaperones and transporters.^17^, ^25^, ^56^ Selection of cells for resistance to cisplatin frequently resulted in cross‐resistance to copper, and similarly, selection for resistance to copper resulted in cross‐resistance to cisplatin. Researches of cells selected for either copper or cisplatin resistance indicate that simultaneous changes occur in the cellular pharmacology of both copper and cisplatin such that cellular accumulation of both is reduced.^46^ Usually, cisplatin‐resistant cell lines have increased expression of either ATP7A or ATP7B. Thus, the enhanced export of cisplatin may be the most crucial contributor to the reduced accumulation of cisplatin, so commonly observed in cisplatin‐resistant cells. Drug resistance is a multifactorial problem. In addition to the factors associated with ATP7A and copper transport, there must be other important mechanisms accounting for the drug resistant phenotype. Furthermore, interaction between cisplatin and ATP7A remains unknown. ATP7A sequestrates cisplatin from its targets as one of the cellular components, or transfers intracellular cisplatin into the vesicular secretory pathway for exporting from cells. Our findings supported the second hypothesis that cisplatin‐treated cells could elevate their vesicular transport systems and accelerate drug release.

Deregulation of miR‐148a‐3p has been declared in various cancers, including pancreatic cancer, ovarian cancer, gastric cancer, lung cancer, and breast cancer.^57^ Moreover, as discussed earlier, numerous targets of miR‐148a‐3p have been identified in breast cancer, including ALCAM, NRP1, SMAD2, and SPIN1, they mainly function in regulating cell growth, metastasis, and adhesion. Meanwhile, we made the finding that in our experiment miR‐148a‐3p targets ATP7A.

MiR‐148a‐3p overexpression significantly enhanced the suppressive effect of cisplatin on breast cancer cell lines. Then, we investigated whether miR‐148a‐3p affected the cisplatin resistance on breast cancer cell lines through reducing ATP7A. Either ATP7A knockdown or miR‐148a overexpression amplified the suppressive effect of cisplatin on cell viability. In addition, CCK‐8 assays showed the effect of miR‐148a‐3p overexpression on cisplatin‐induced cell proliferation suppression could be partially reversed by overexpressed ATP7A. However, we need more clinical samples to further confirm the above findings, which should be addressed in our future studies.

## 5 CONCLUSIONS

In summary, the present study identified copper efflux transporter ATP7A, as a novel target of miR‐148a‐3p, played a critical role in resistance of breast cancer cells to cisplatin via sequestering and pumping cisplatin out of cancer cells. As cisplatin is widely used for breast cancer therapy, and cisplatin resistance is a common problem associated with chemotherapy, our discovery provides useful information for the treatment strategies of drug‐resistant breast cancer patients.

## CONFLICT OF INTEREST

The authors declare that there is no conflict of interest that could be perceived as prejudicing the impartiality of the research reported.

## AUTHOR CONTRIBUTIONS

Ze Yu designed and performed experiments, analyzed data, and wrote the paper. Weifan Cao performed miRNA binding experiments, characterization of formulations and RT‐PCR experiments. Yuan Ren was involved in RT‐PCR data analysis and figure preparation. Qijia Zhang participated in method section writing. Jia Liu conceived the idea, analyzed data, and manuscript editing. All authors read and approved the final manuscript.

## FUNDING INFORMATION

The present study was funded by Heilongjiang Province Scientific Research Institution Innovation Grants (YC2016D002).

## AVAILABILITY OF DATA AND MATERIALS

The data in the current study are based on public data available, and some data used during the study appear in the submitted Supplementary Material and Figure legends. All data that support the findings of this study are available from the corresponding author upon reasonable request.

## ETHICS APPROVAL AND CONSENT TO PARTICIPATE

All animals received humane care according to the criteria outlined in the Guide for the Care and Use of Laboratory Animals. All animal experiments were conducted in accordance with the guidelines of the Animal Care and Ethics Committee of Guangzhou Medical University.

## Supporting information

Supplement Information.

Supplement Information.

Supplement Information.

Supplement Information.

Supplement Information.

Supplement Information.

## References

[1] Anastasiadi Z , Lianos GD , Ignatiadou E , Harissis HV , Mitsis M . Breast cancer in young women: an overview. Updates Surg. 2017;69:313‐317. 28260181 10.1007/s13304-017-0424-1

[2] Colditz GA , Bohlke K . Priorities for the primary prevention of breast cancer. CA Cancer J Clin. 2014;64:186‐194. 24647877 10.3322/caac.21225

[3] Majidinia M , Yousefi B . DNA repair and damage pathways in breast cancer development and therapy. DNA Repair (Amst). 2017;54:22‐29. 28437752 10.1016/j.dnarep.2017.03.009

[4] Weitzel JN . The genetics of breast cancer: what the surgical oncologist needs to know. Surg Oncol Clin N Am. 2015;24:705‐732. 26363538 10.1016/j.soc.2015.06.011

[5] Fan L , Strasser‐Weippl K , Li JJ , et al. Breast cancer in China. Lancet Oncol. 2014;15: e279‐e289. 24872111 10.1016/S1470-2045(13)70567-9

[6] Narod SA , Salmena L . BRCA1 and BRCA2 mutations and breast cancer. Discov Med. 2011;12:445‐453. 22127115

[7] Woolston C . Breast cancer. Nature. 2015;527: S101. 26580154 10.1038/527S101a

[8] Al‐Hilli Z , Boughey JC . The timing of breast and axillary surgery after neoadjuvant chemotherapy for breast cancer. Chin Clin Oncol. 2016;5:37. 27164853 10.21037/cco.2016.03.26

[9] Yang M , Li Y , Shen X , et al. CLDN6 promotes chemoresistance through GSTP1 in human breast cancer. J Exp Clin Cancer Res. 2017;36:157. 29116019 10.1186/s13046-017-0627-9 PMC5678781

[10] Fan X , Zhou S , Zheng M , et al. MiR‐199a‐3p enhances breast cancer cell sensitivity to cisplatin by downregulating TFAM (TFAM). Biomed Pharmacother. 2017;88:507‐514. 28126676 10.1016/j.biopha.2017.01.058

[11] Silver DP . Efficacy of neoadjuvant cisplatin in triple‐negative breast cancer. J Clin Oncol. 2010;28:1145‐1153. 20100965 10.1200/JCO.2009.22.4725 PMC2834466

[12] Dasari S , Tchounwou PB . Cisplatin in cancer therapy: molecular mechanisms of action. Eur J Pharmacol. 2014;740:364‐378. 25058905 10.1016/j.ejphar.2014.07.025 PMC4146684

[13] Galluzzi L , Senovilla L , Vitale I , et al. Molecular mechanisms of cisplatin resistance. Oncogene. 2012;31: 1869‐1883. 21892204 10.1038/onc.2011.384

[14] Kartalou M , Essigmann JM . Mechanisms of resistance to cisplatin. Mutat Res. 2001;478:23‐43. 11406167 10.1016/s0027-5107(01)00141-5

[15] Eljack ND , Ma HY , Drucker J , et al. Mechanisms of cell uptake and toxicity of the anticancer drug cisplatin. Metallomics. 2014;6:2126‐2133. 25306996 10.1039/c4mt00238e

[16] Yue Z , Cao Z . Current Strategy for Cisplatin Delivery. Curr Cancer Drug Targets. 2016;16:480‐488. 27018267 10.2174/1568009616666160328113006

[17] Huang CP , Fofana M , Chan J , Chang CJ , Howell SB . Copper transporter 2 regulates intracellular copper and sensitivity to cisplatin. Metallomics. 2014;6:654‐661. 24522273 10.1039/c3mt00331k PMC3982597

[18] Helleman J , Burger H , Hamelers IH , et al. Impaired cisplatin influx in an A2780 mutant cell line: evidence for a putative, cis‐configuration‐specific, platinum influx transporter. Cancer Biol Ther. 2006;5:943‐949. 16775422 10.4161/cbt.5.8.2876

[19] Amable L . Cisplatin resistance and opportunities for precision medicine. Pharmacol Res. 2016;106:27‐36. 26804248 10.1016/j.phrs.2016.01.001

[20] Samimi G , Varki NM , Wilczynski S , Safaei R , Alberts DS , Howell SB . Increase in expression of the copper transporter ATP7A during platinum drug‐based treatment is associated with poor survival in ovarian cancer patients. Clin Cancer Res. 2003;9:5853‐5859. 14676106

[21] Telianidis J , Hung YH , Materia S , Fontaine SL . Role of the P‐Type ATPases, ATP7A and ATP7B in brain copper homeostasis. Front Aging Neurosci. 2013;5:44. 23986700 10.3389/fnagi.2013.00044 PMC3750203

[22] Li YQ , Yin JY , Liu ZQ , Li XP . Copper efflux transporters ATP7A and ATP7B: Novel biomarkers for platinum drug resistance and targets for therapy. IUBMB Life. 2018;70:183‐191. 29394468 10.1002/iub.1722

[23] Tadini‐Buoninsegni F , Bartolommei G , Moncelli MR , et al. Translocation of platinum anticancer drugs by human copper ATPases, ATP7A and ATP7B. Angew Chem Int Ed Engl. 2014;53:1297‐1301. 24375922 10.1002/anie.201307718 PMC3937162

[24] Komatsu M . Copper‐transporting P‐type adenosine triphosphatase (ATP7B) is associated with cisplatin resistance. Cancer Res. 2000;60:1312‐1316. 10728692

[25] Zhu S , Shanbhag V , Wang Y , Lee J , Petris M . A role for the atp7a copper transporter in tumorigenesis and cisplatin resistance. J Cancer. 2017;8:1952‐1958. 28819394 10.7150/jca.19029 PMC5559955

[26] Inoue Y , Matsumoto H , Yamada S et al. ATP7B expression is associated with in vitro sensitivity to cisplatin in nonsmall cell lung cancer. Oncol Lett. 2010;1:279‐282. 22966294 10.3892/ol_00000049 PMC3436352

[27] Ambros V . The functions of animal microRNAs. Nature. 2004;431:350‐355. 15372042 10.1038/nature02871

[28] Xu X , Zhang Y , Jasper J , et al. MiR‐148a functions to suppress metastasis and serves as a prognostic indicator in triple‐negative breast cancer. Oncotarget. 2016;7:20381‐20394. 26967387 10.18632/oncotarget.7953 PMC4991462

[29] Jiang F , Li Y , Mu J , et al. Glabridin inhibits cancer stem cell‐like properties of human breast cancer cells: an epigenetic regulation of miR‐148a/SMAd2 signaling. Mol Carcinog. 2016;55:929‐940. 25980823 10.1002/mc.22333

[30] Li B , Wang W , Li Z , et al. MicroRNA‐148a‐3p enhances cisplatin cytotoxicity in gastric cancer through mitochondrial fission induction and cyto‐protective autophagy suppression. Cancer Lett. 2017;1:212‐227. 10.1016/j.canlet.2017.09.035 PMC5675767 28965855

[31] Yu Z , Zhou R , Zhao Y , et al. Blockage of SLC31A1‐dependent copper absorption increases pancreatic cancer cell autophagy to resist cell death. Cell Prolif. 2019;52: e12568. 30706544 10.1111/cpr.12568 PMC6496122

[32] Bai F , Yu Z , Gao X , Gong J , Fan L , Liu F . Simvastatin induces breast cancer cell death through oxidative stress up‐regulating miR‐140‐5p. Aging (Albany NY). 2019;11:3198‐3219. 31138773 10.18632/aging.101974 PMC6555469

[33] Li JH , Liu S , Zhou H , Qu LH , Yang JH . starBase v2.0: decoding miRNA‐ceRNA, miRNA‐ncRNA and protein‐RNA interaction networks from large‐scale CLIP‐Seq data. Nucleic Acids Res. 2014;42: D92‐D97. 24297251 10.1093/nar/gkt1248 PMC3964941

[34] Gao J , Aksoy BA , Dogrusoz U , et al. Integrative analysis of complex cancer genomics and clinical profiles using the cBioPortal. Sci Signal. 2013;6(269)pl1.. 23550210 10.1126/scisignal.2004088 PMC4160307

[35] Vasaikar SV , Straub P , Wang J , Zhang B . LinkedOmics: analyzing multi‐omics data within and across 32 cancer types. Nucleic Acids Res. 2018;46: D956‐D963. 29136207 10.1093/nar/gkx1090 PMC5753188

[36] Tang Z , Li C , Kang B , Gao G , Li C , Zhang Z . GEPIA: a web server for cancer and normal gene expression profiling and interactive analyses. Nucleic Acids Res. 2017;45:W98‐W102. 28407145 10.1093/nar/gkx247 PMC5570223

[37] Zweig AS , Karolchik D , Kuhn RM , Haussler D , Kent WJ . UCSC genome browser tutorial. Genomics. 2008;92:75‐84. 18514479 10.1016/j.ygeno.2008.02.003

[38] Nagy Á , Lánczky A , Menyhárt O , Győrffy B . Validation of miRNA prognostic power in hepatocellular carcinoma using expression data of independent datasets. Sci Rep. 2018;15:9227. 10.1038/s41598-018-27521-y PMC6003936 29907753

[39] Aguirre‐Gamboa R , Gomez‐Rueda H , Martínez‐Ledesma E , et al. SurvExpress: an online biomarker validation tool and database for cancer gene expression data using survival analysis. PLoS One. 2013;8:e74250. 24066126 10.1371/journal.pone.0074250 PMC3774754

[40] Song X , Zhao C , Jiang L , et al. High PITX1 expression in lung adenocarcinoma patients is associated with DNA methylation and poor prognosis. Pathol Res Pract. 2018;214:2046‐2053. 30322808 10.1016/j.prp.2018.09.025

[41] Barretina J , Caponigro G , Stransky N , et al. The Cancer Cell Line Encyclopedia enables predictive modelling of anticancer drug sensitivity. Nature. 2012;483:603‐607. 22460905 10.1038/nature11003 PMC3320027

[42] Li J , Zhao W , Akbani R , et al. Characterization of human cancer cell lines by reverse‐phase protein arrays. Cancer Cell. 2017;31:225‐239. 28196595 10.1016/j.ccell.2017.01.005 PMC5501076

[43] Safaei R , Katano K , Samimi G , et al. Cross‐resistance to cisplatin in cells with acquired resistance to copper. Cancer Chemother Pharmacol. 2004;53:239‐246. 14648017 10.1007/s00280-003-0736-3

[44] Dolgova NV , Nokhrin S , Yu CH , George GN , Dmitriev OY . Copper chaperone Atox1 interacts with the metal binding domain of Wilson's disease protein in cisplatin detoxification. Biochem J. 2013;454:147‐156. 23751120 10.1042/BJ20121656

[45] Li T , Peng J , Zeng F , et al. Association between polymorphisms in CTR1, CTR2, ATP7A, and ATP7B and platinum resistance in epithelial ovarian cancer. Int J Clin Pharmacol Ther. 2017;55:774‐780. 28737129 10.5414/CP202907

[46] Kilari D , Guancial E , Kim ES . Role of copper transporters in platinum resistance. World J Clin Oncol. 2016;7:106‐113. 26862494 10.5306/wjco.v7.i1.106 PMC4734932

[47] Udhayakumari D , Velmathi S , Sung YM , Wu SP . Highly fluorescent probe for copper (II) ion based on commercially available compounds and live cell imaging. Sensor Actuat B‐Chem. 2014;198:285‐293.

[48] Chen MJ , Cheng YM , Chen CC , Chen YC , Shen CJ . MiR‐148a and miR‐152 reduce tamoxifen resistance in ER+ breast cancer via downregulating ALCAM. Biochem Biophys Res Commun. 2017;483:840‐846. 28063929 10.1016/j.bbrc.2017.01.012

[49] Chen X , Wang YW , Gao P . SPIN1, negatively regulated by miR‐148/152, enhances Adriamycin resistance via upregulating drug metabolizing enzymes and transporter in breast cancer. J Exp Clin Cancer Res. 2018;37:100. 29743122 10.1186/s13046-018-0748-9 PMC5944004

[50] Li ZH , Zheng R , Chen JT , Jia J , Qiu M . The role of copper transporter ATP7A in platinum‐resistance of esophageal squamous cell cancer (ESCC). J Cancer. 2016;7:2085‐2092. 27877224 10.7150/jca.16117 PMC5118672

[51] Pauwels EK , Erba P , Mariani G , Gomes CM . Multidrug resistance in cancer: its mechanism and its modulation. Drug News Perspect. 2007;20:371‐377. 17925891 10.1358/dnp.2007.20.6.1141496

[52] Chen X , Lu P , Wu Y , et al. MiRNAs‐mediated cisplatin resistance in breast cancer. Tumour Biol. 2016;37:12905‐12913. 27448297 10.1007/s13277-016-5216-6

[53] Siddik ZH . Cisplatin: mode of cytotoxic action and molecular basis of resistance. Oncogene. 2003;22:7265‐7279. 14576837 10.1038/sj.onc.1206933

[54] Chisholm CL , Wang H , Wong AH , et al. Ammonium tetrathiomolybdate treatment targets the copper transporter ATP7A and enhances sensitivity of breast cancer to cisplatin. Oncotarget. 2016;7:84439‐84452. 27806319 10.18632/oncotarget.12992 PMC5341295

[55] Xiao F , Li Y , Wan Y , Xue M . MircroRNA‐139 sensitizes ovarian cancer cell to cisplatin‐based chemotherapy through regulation of ATP7A/B. Cancer. Chemother Pharmacol. 2018;81:935‐947. 29594361 10.1007/s00280-018-3548-1

[56] Akerfeldt MC , Tran CM , Shen C , Hambley TW , New EJ . Interactions of cisplatin and the copper transporter CTR1 in human colon cancer cells. J Biol Inorg Chem. 2017;22:765‐774. 28516214 10.1007/s00775-017-1467-y

[57] Li Y , Deng X , Zeng X , Peng X . The Role of Mir‐148a in Cancer. J Cancer. 2016;7:1233‐1241. 27390598 10.7150/jca.14616 PMC4934031

