# Supplementary material for: ATPase copper transporter A, negatively regulated by miR‐148a‐3p, contributes to cisplatin resistance in breast cancer cells
Source: Clin Transl Med. 2020 Apr 7;10(1):57–73. doi: 10.1002/ctm2.19 (PMC7240853; doi:10.1002/ctm2.19)
Supplement: Supplementary file 1 — Supplement Information. [file CTM2-10-57-s006.docx]

**Supplementary Material**

**siRNA sequences:** Design of siRNA: siDirect version 2.0 **(**[**http://sidirect2.rnai.jp/**](http://sidirect2.rnai.jp/)**)**

& Designer of Small Interfering RNA **(http://biodev.extra.cea.fr/DSIR/DSIR.php)**

1. **ATP7A**

5’- UAUCCUAUGGUUAAACCUCUG-3’; 5’- GAGGUUUAACCAUAGGAUAGA-3’

1. **ALCAM**

5’- GAAUACAAAGACAGAUUGAAC-3’; 5’- UCAAUCUGUCUUUGUAUUCUG-3’

1. **NRP1**

5’- UUGAAGUUGAUCAUAAUUCUC-3’; 5’- GAAUUAUGAUCAACUUCAACC-3’

1. **SMAD2**

5’- GUGUUACCAUACCAAGAUAUU-3’; 5’- UAUCUUGGUAUGGUAACACAU-3’

1. **SPIN1**

5’- AGAACUUAAUAAAGAUGAAAG-3’; 5’- UUCAUCUUUAUUAAGUUCUAG-3’

**Primer sequences:**

1. **ATP7A**

Up: 5’- UAUCCUAUGGUUAAACCUCUG-3’; Down: 5’- AGGUUUAACCAUAGGAUAGA-3’

1. **ALCAM**

Up: 5’- ACTTGACGTACCTCAGAATCTCA-3’; Down: 5’- CATCGTCGTACTGCACACTTT-3’

1. **NRP1**

Up: 5’- ACGTGGAAGTCTTCGATGGAG-3’; Down: 5’- CACCATGTGTTTCGTAGTCAGA-3’

1. **SMAD2**

Up: 5’- CGTCCATCTTGCCATTCACG-3’; Down: 5’- CTCAAGCTCATCTAATCGTCCTG-3’

1. **SPIN1**

Up: 5’- GAGGGGAATGGCCCTGTTAC-3’; Down: 5’- GACTTCAAGCGCAGAAACTCT-3’

**Bioinformatics Database websites:**

1. Cisplatin-induced gene expression changes in triple-negative breast cancer (TNBC) cells (**GSE103115**); miRNAs expression in breast cancer tumor tissues with or without axillary lymph nodes metastasis (**GSE100453**); has-miR-148a expression levels in cancer cells (**GSE86195, GSE79506, GSE54665, GSE93795 and GSE84200**) were obtained from the GEO Database: https://www.ncbi.nlm.nih.gov/gds/

Relative gene and miRNA expression levels in breast cancer: tumor (**GDS4069**) were obtained from GEO Profiles: <https://www.ncbi.nlm.nih.gov/geoprofiles/>

1. Functional Categories and Gene Ontology analysis (**GSE103115**) were performed through the online database **DAVID**: <https://david.ncifcrf.gov/>
2. Correlation analysis of gene expression (RNA-RNA Co-Expression) were obtained via the **ENCORI** Pan-Cancer Analysis Platform website. & The correlation of miR-148a-3p and miR-148b-3p levels and the survival time was analyzed using data from the **ENCORI** database: <http://starbase.sysu.edu.cn/panCancer.php>
3. The expression levels of ATP7A/ATP7B in various tumors were obtained via the **GEPIA** website: Gene Expression Profiling Interactive Analysis.& The correlation of ATP7A and ATP7B mRNA levels and the survival time was analyzed using data from the **GEPIA** database: <http://gepia.cancer-pku.cn/>
4. Prediction of microRNA targets: targetscan, TarBase v.8, miranda and miRDB

**Targetscan:** http://www.targetscan.org/vert_72/

**TarBase v.8**: <http://carolina.imis.athena-innovation.gr/diana_tools/>

**Miranda:** <http://miranda.org.uk/>; **miRDB:** http://mirdb.org/

1. the level of has-miR-148a-3p was compared with has-miR-148a-5p. This result was from the **YM500v2** miRNA database: <http://ngs.ym.edu.tw/ym500v2/index.php>
2. Correlation analysis, survival curves analysis, enrichment analysis (heatmap and GO analysis) of ATP7A/7B and miR-148a-3p negatively correlated genes (RNAseq & Proteome) all came from **linkedOmics**: <http://www.linkedomics.org/login.php>
3. Correlation analysis of ATP7A and ATP7B came from **cBioPortal**:

<https://www.cbioportal.org/>

1. Survival curves of ATP7A and ATP7B and Correlation analysis between methylation and expression of ATP7A were from the **UCSC Xena**: <http://xena.ucsc.edu/>
2. Survival curves were analyzed using data from the **KMplot**: <http://kmplot.com/>
3. Survival biomarkers analysis (ATP7A/ATP7B) for cancer outcomes, SurvExpress: <http://bioinformatica.mty.itesm.mx:8080/Biomatec/SurvivaX.jsp>
4. CCLE provides the expression levels of ATP7A and miR-148a-3p in breast cancer cells: <https://portals.broadinstitute.org/ccle/about#terms>
5. Protein-drug Correlation Analysis dataset was obtained from TCPA-portal (MCLP):

<https://tcpaportal.org/mclp/#/>

**Supplementary Figure legends**

**Additional file Figure S1.** Enrichment analysis of Positively Correlated Genes with ATP7A and ATP7B. (A) Positively Correlated significant Genes with ATP7A. (B) ATP7A association result. (C) Bar chart of Biological, Cellular Component and Molecular Function categories with ATP7A. (D&E) Gene Ontology analysis with ATP7A. (F) Positively Correlated significant Genes with ATP7B. (G) ATP7B association result. (H) Bar chart of Biological, Cellular Component and Molecular Function categories with ATP7B. (I&J) Gene Ontology analysis with ATP7B.

**Additional file Figure S2.** Knocking-down ATP7B did not contribute to cisplatin resistance. (A) Flow cytometry analysis was performed to detect the apoptosis in cisplatin-treated MDA-MB-231 cells transfected with si-ATP7B or si-nc for 48 hours. (B) Bubble chart showing the top enriched pathways by DAVID enrichment (GO analysis) to demonstrate the difference in gene enrichment in cisplatin-induced TNBC cells. (C) qRT-PCR analysis of ATP7A expression in MDA-MB-231 cell treated with 5μM or 10μM cisplatin. All the results are presented as the means ± SD of values obtained in three independent experiments, *P<0.05; ***P<0.001.

**Additional file Figure S3.** Copper promotes cisplatin resistance in breast cancer cells. (A, B) IF and western blot were used to detect cisplatin-induced caspase-3-dependent apoptosis in MDA-MB-231 and T47D cells under TM treatment, compared with cisplatin alone. (C) MDA-MB-231 cells were treated with different doses of cisplatin in the presence of CuSO_4_ or TM for 48 hours, and assessed for cell viability by CCK-8 assay. (D) Schematic model of the role of copper in breast cancer cell cisplatin-resistance. All the results are presented as the means ± SD of values obtained in three independent experiments, *P<0.05; **P<0.01.

**Additional file Figure S4.** The expression level of miR-148a is lower than that of miR-148b in breast cancer cells and samples. (A, B) Expression of miR-148a-3p vs miR-148a-5p in human tissues, the data came from YM500 miRNA database. (C) Relative expression levels of miRNAs in breast cancer tissues with or without axillary lymph nodes metastasis. (D) Relative expression levels of miR-148a and miR-148b in MDA-MB-231 and T47D cells were determined using RT-PCR. (E) Relative expression levels of miR-148a and miR-148b in breast cancer samples (TNBC, n=5; Non-TNBC, n=14) were obtained from GEO Profiles. All the results are presented as the means ± SD of values obtained in three independent experiments, **P<0.01; ***P<0.001.

**Additional file Figure S5.** MiR-148a-3p promotes cisplatin-sensitivity in breast cancer cells through suppressing ATP7A. (A) cell viability assay of T47D cells treated with miR-148a-3p. (B) Caspase-3 protein of MDA-MB-231 cells transfected with miR-148a-3p mimic was measured by western blot analysis. (C) The effect of miR-148a-3p on apoptosis was evaluated by flow cytometry using Annexin V/PI staining kit. (D) western blot analysis of caspase-3 was used to detect apoptosis in each group.
