# Supplementary figures and images for: ATPase copper transporter A, negatively regulated by miR‐148a‐3p, contributes to cisplatin resistance in breast cancer cells
Source: Clin Transl Med. 2020 Apr 7;10(1):57–73. doi: 10.1002/ctm2.19 (PMC7240853; doi:10.1002/ctm2.19)

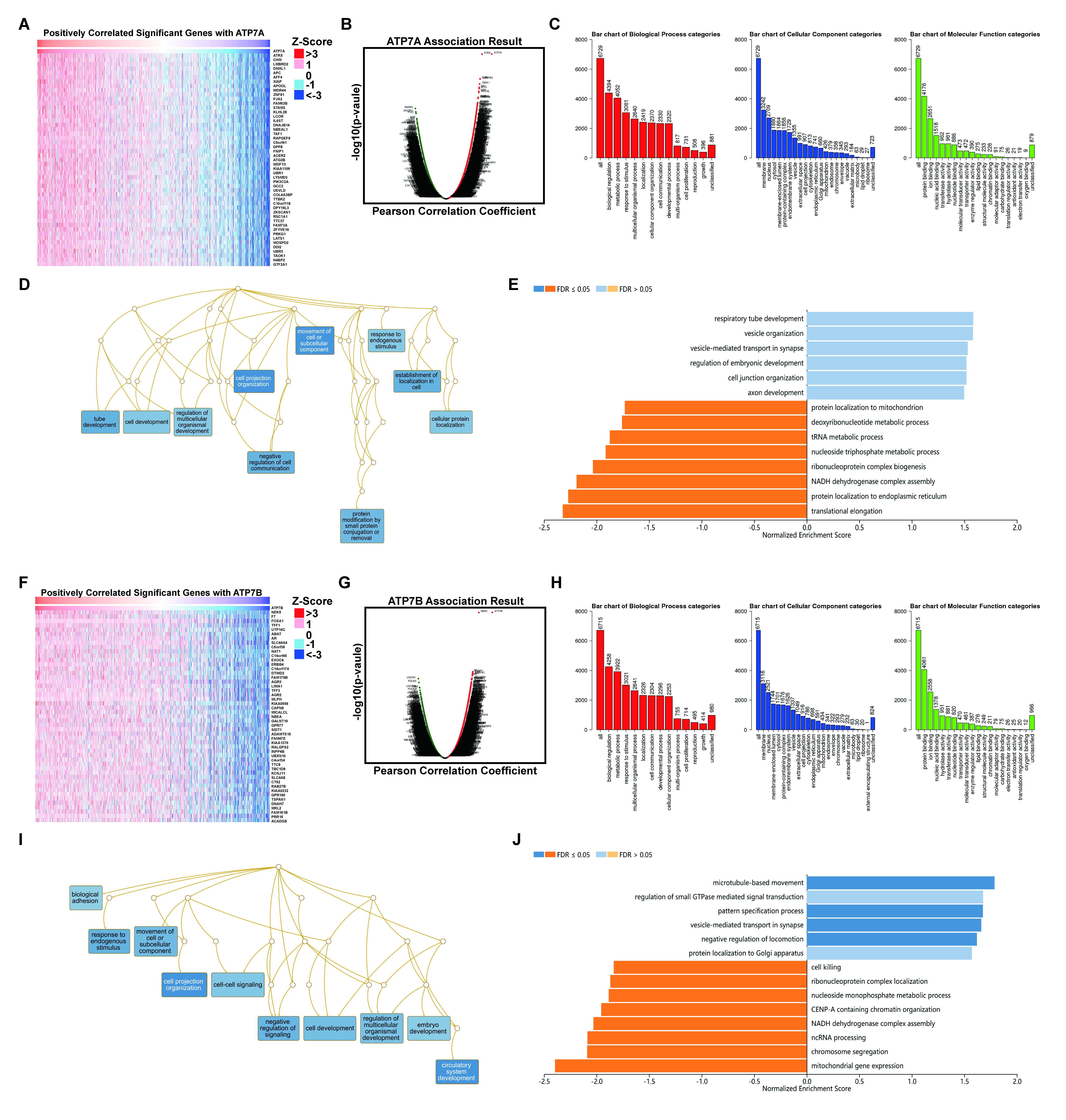

Supplement: Supplementary file 2 — Supplement Information. [file CTM2-10-57-s001.tif]

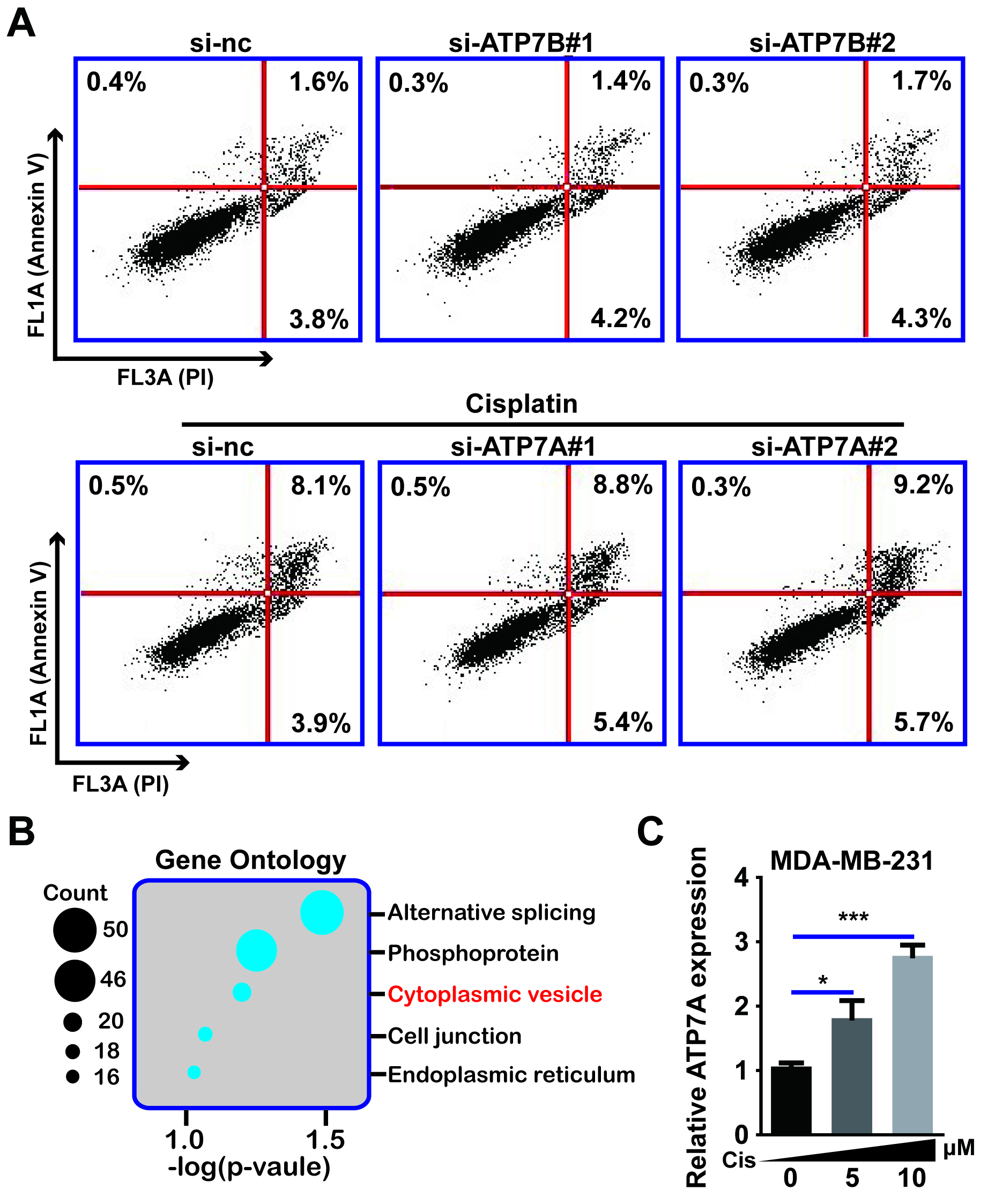

Supplement: Supplementary file 3 — Supplement Information. [file CTM2-10-57-s002.tif]

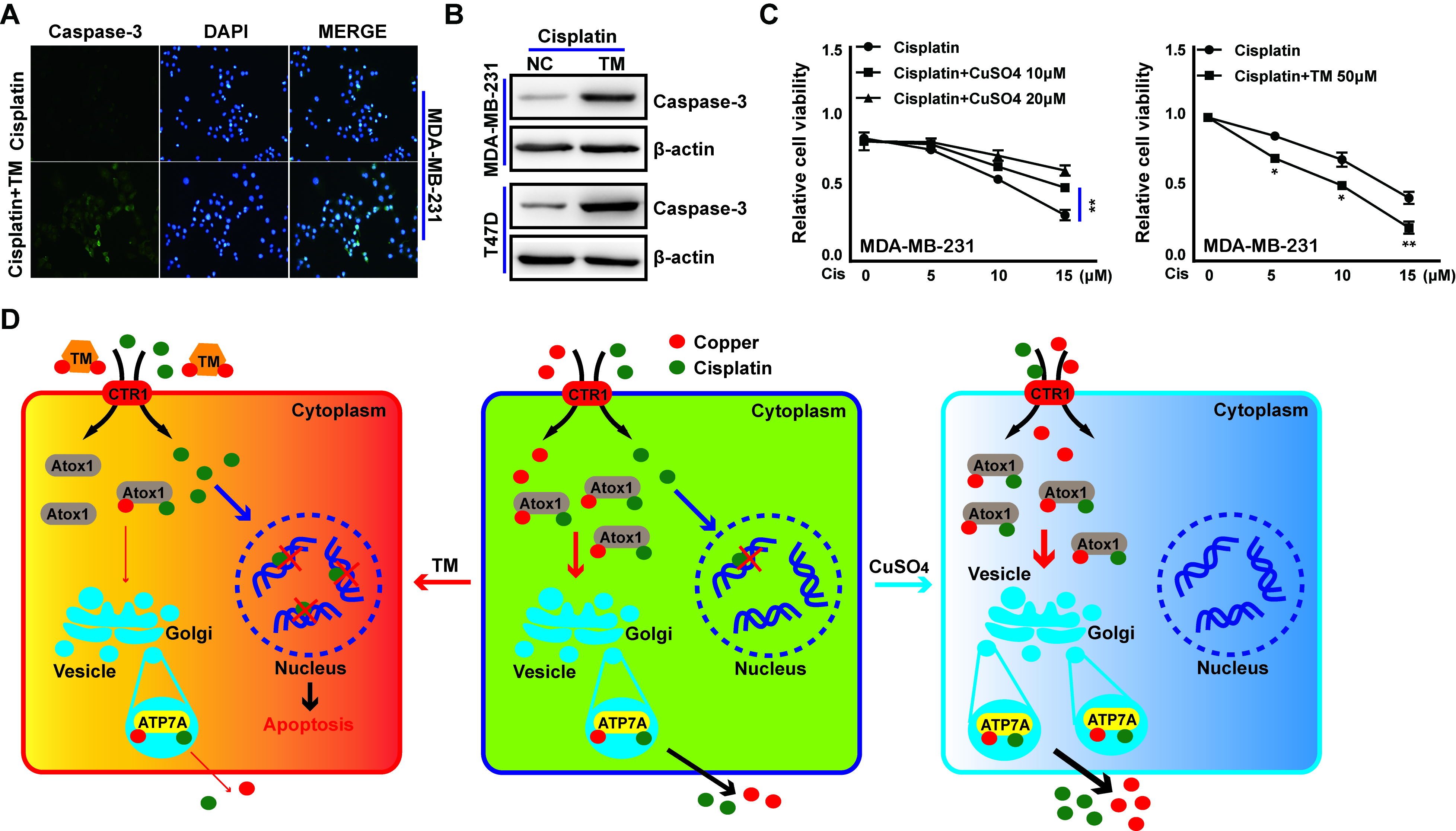

Supplement: Supplementary file 4 — Supplement Information. [file CTM2-10-57-s003.tif]

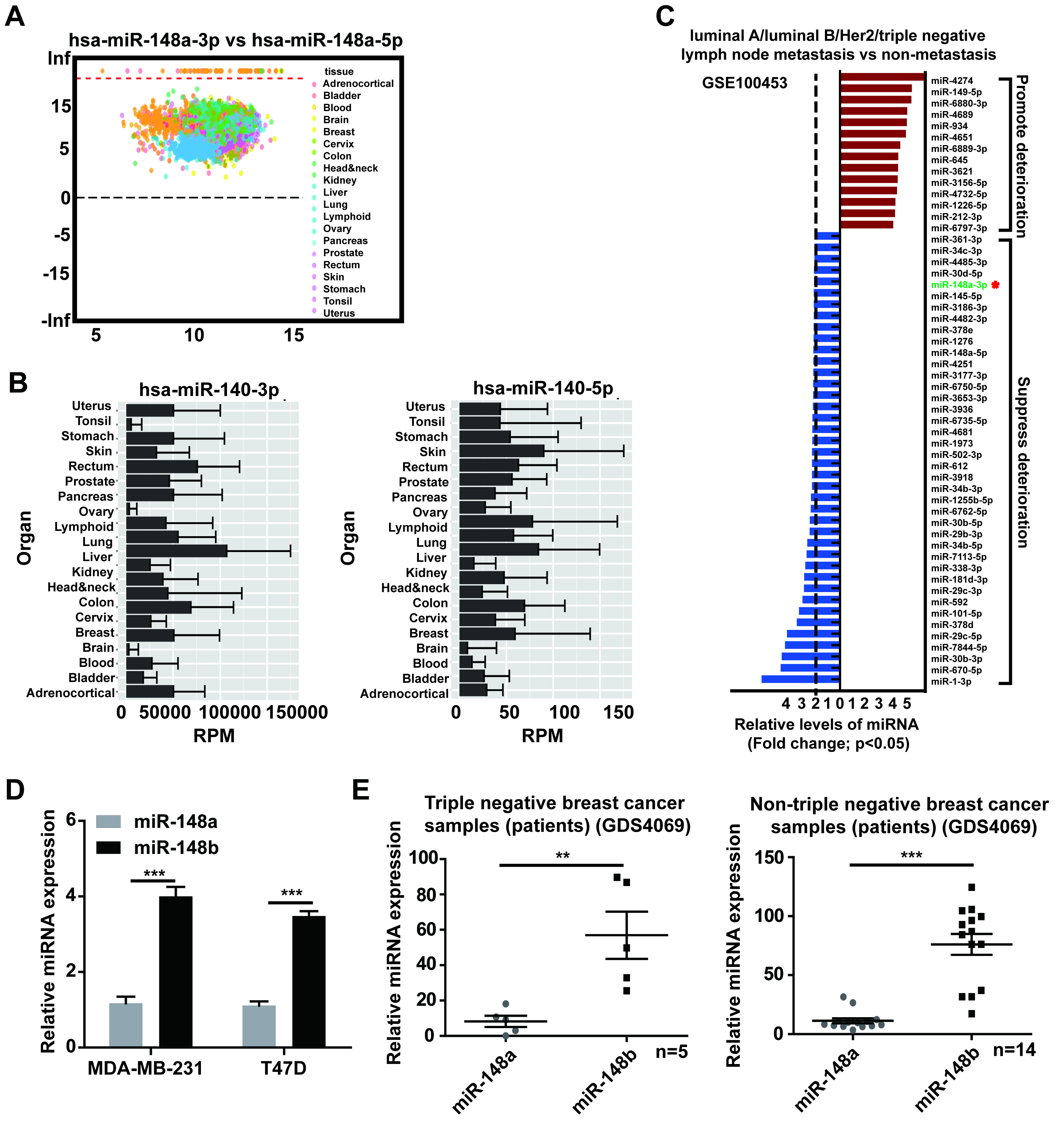

Supplement: Supplementary file 5 — Supplement Information. [file CTM2-10-57-s004.tif]

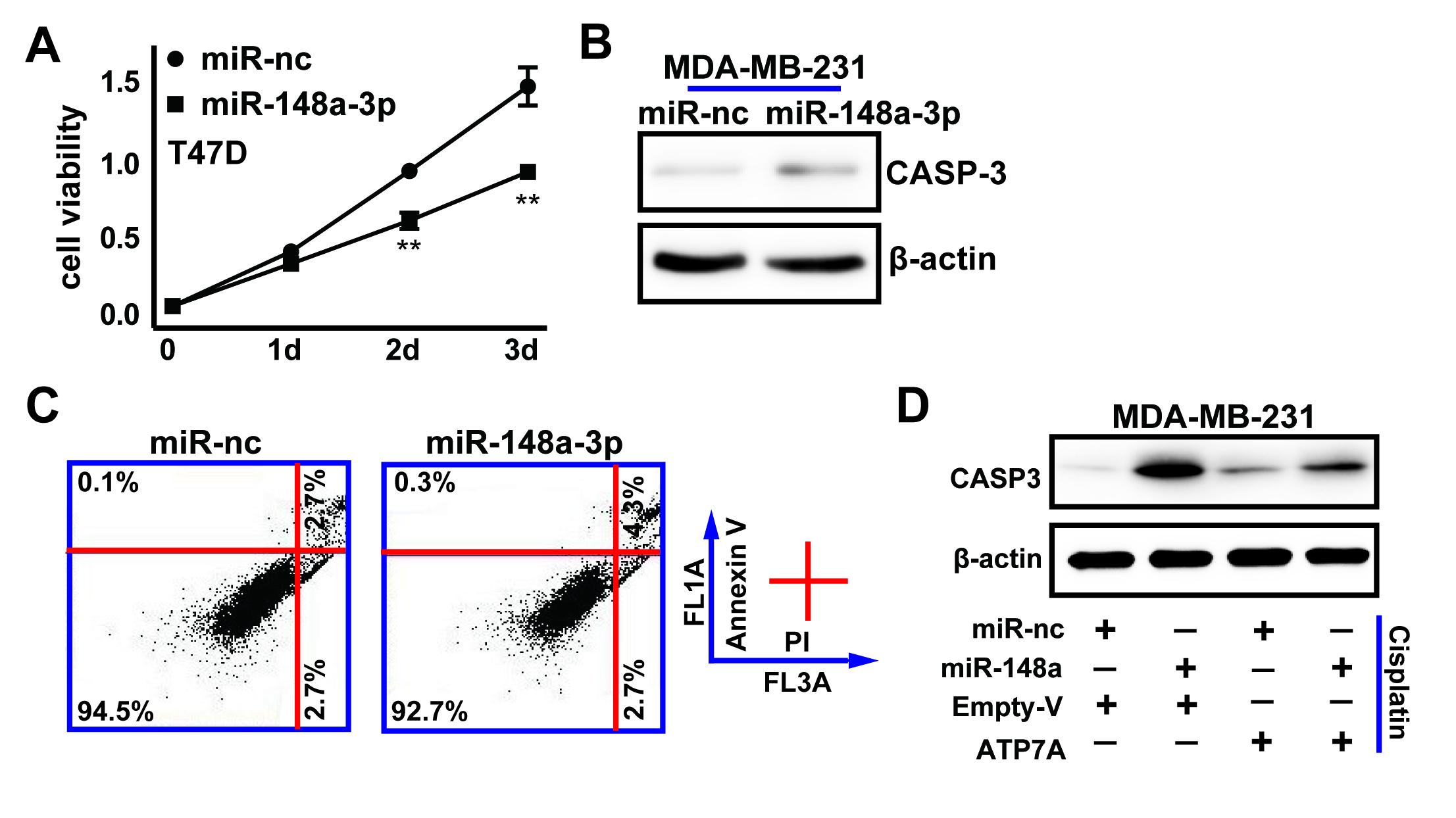

Supplement: Supplementary file 6 — Supplement Information. [file CTM2-10-57-s005.tif]
